# Dual-Mode Induction of Tunable Circularly Polarized Luminescence from Chiral Metal-Organic Frameworks

**DOI:** 10.34133/2020/6452123

**Published:** 2020-01-23

**Authors:** Tonghan Zhao, Jianlei Han, Xue Jin, Minghao Zhou, Yan Liu, Pengfei Duan, Minghua Liu

**Affiliations:** ^1^CAS Center for Excellence in Nanoscience, CAS Key Laboratory of Nanosystem and Hierarchical Fabrication, National Center for Nanoscience and Technology (NCNST), No. 11, ZhongGuanCun BeiYiTiao, 100190 Beijing, China; ^2^University of Chinese Academy of Sciences, Beijing 100049, China; ^3^School of Chemistry and Chemical Engineering, Shanghai Jiao Tong University, Shanghai 200240, China; ^4^Beijing National Laboratory for Molecular Science, CAS Key Laboratory of Colloid, Interface and Chemical Thermodynamics, Institute of Chemistry, Chinese Academy of Sciences, No. 2, ZhongGuanCun BeiYiJie, Beijing 100190, China

## Abstract

The general approach for fabricating solid-state materials showing circularly polarized luminescence (CPL) is still in its challenge. In this work, chiral metal-organic frameworks (MOFs) with full-color and white-color circularly polarized light emission are firstly achieved through a host-guest emitter-loading strategy. Chiral zeolitic imidazolate frameworks (ZIFs, a class of MOFs) are fabricated by a facile and simple mixed-ligand coassembly pathway. Meantime, achiral dyes, quantum dots (QDs), and upconversion nanoparticles (UCNPs) are easily loaded into the chiral ZIFs during the synthetic process. Size-matched dyes can be solely encapsulated into the chiral cages of ZIF, resulting in induced CPL and enhanced luminescence efficiency in solid-state ZIF⊃dye composites. Large-sized QDs, after embedding into the gap of the ZIF particles, also exhibited intense CPL activity. Furthermore, through modulating the blending ratio of colored dyes or QDs in chiral ZIFs, white light-emitting ZIFs with circular polarization could be constructed in a solid state. In addition, through loading rare earth element-based upconversion nanoparticles (UCNPs) into chiral ZIFs, upconverted CPL (UC-CPL) could be achieved with a high dissymmetry factor (*g*_lum_). Thus, various achiral luminophores were endowed with CPL upon coupling with chiral ZIFs, which significantly deepened and enlarged the research scope of the chiroptical materials in a solid state.

## 1. Introduction

Chiral functional materials with circularly polarized luminescence (CPL) have risen to prominence in recent years due to their fascinating properties with wide potential applications in chiral sensors [1, 2], photoelectric devices [3–5], 3D optical displays [6, 7], encrypted storage of information [8, 9], and even dissymmetric synthesis [10–12]. The general approach to fabricate CPL-active materials is through a covalent bonding of luminophore with a chiral moiety [13–15]. Varieties of chiral moieties incorporated with luminophores by covalent bonds, such as chiral polymers [16–18], chiral metal complexes [19, 20], and chiral liquid crystals [21, 22], have been widely investigated as CPL candidates. However, the CPL properties of obtained chiral materials are sometimes unpredictable, and a tedious synthesis process is always inevitable [23, 24]. In this context, the combination of chiral host with achiral luminescent guests through chirality transfer to fabricate CPL-active materials recently becomes an arising approach [25–31].

To endow achiral luminophores with CPL activity, an attracting approach is introducing the achiral luminescent guest emitter to a chiral host. Considering that the emitters are achiral, the obtained CPL signal is ascribed to the induced chirality through chirality transfer from a chiral matrix to achiral guest emitters. For the chirality transfer, the noncovalent interactions between the chiral host matrix and the guest emitters are of utmost importance. The design of molecules with suitable bonding sites and their cooperation play a very important role. For example, Goto et al. have reported a class of charged amphiphilic molecules which could form chiral nanofibrillar aggregations in aqueous solutions [25]. Anionic dyes could coassemble with the chiral template by electrostatic interaction, exhibiting intense circularly polarized emission. A similar approach involving noncovalent interaction for fabricating CPL-active materials has been demonstrated including emissive chalcogenide semiconductor quantum dots (QDs) and all-inorganic perovskite nanocrystals [26–28].

Besides the noncovalent interaction, confined chiral space has been reported as a versatile chiral host for endowing achiral emitters with CPL activity through a host-guest encapsulation process. For example, chiral *C*_3_ symmetric glutamate-derivative gelator could individually form hexagonal nanotube structures [29]. Achiral dyes, without any bonding sites, could simultaneously aggregate into the confined chiral nanotubes *via* a cogelatinization process, enabling color tunable CPL activity. Considering that the chiral template molecules still require elaborate design and tedious synthesis in the system, developing a simple and versatile chiral host matrix without tedious synthesis should be one of the most important issues in this research field. In addition, most of the reported cases involve a solution phase which definitely limited the practical applications [30, 31]. It is emergent to develop solid-state CPL-active materials by a general approach.

Metal-organic frameworks (MOFs) have received extensive attention due to their wide applications in gas separation and storage, catalysis, delivery, sensing, and so on [32, 33]. Compared with other porous materials, MOFs have special applications as host matrixes due to the unique features, including high crystallinity, regular and tunable structures, ultrahigh porosities and surface areas, and tunable and modifiable pores. In addition to the encapsulation of gas and solvent molecules, different large molecules such as dyes can be incorporated into MOFs with tunable sizes. Due to the good compatibility of MOFs, even large-sized QDs and nanoparticles could be encapsulated into MOFs [34–36]. Up to now, a large number of luminescent MOFs have been reported through loading guest emitters into host MOF matrixes. Meanwhile, many applications such as multiphoton-pumped lasing [37], white light-emitting diodes (WLEDs) [38], second-order nonlinear optics [39], and temperature sensing have been achieved [40]. The utilization of the host-guest chemistry with the MOFs is rising fast, which opens up new avenues for developing advanced multifunctional materials. However, employing chiral MOFs as hosts to endow achiral emitters with CPL activity has never been achieved. Chiral MOFs have attracted great attention because of their potential applications in enantioselective separation [41], catalysis [42–45], and so forth [46, 47]. In addition, chiral MOFs with suitable pores and tunable structures also have potential for loading luminophores. Thus, it is potential to achieve CPL-active materials composed of achiral emitters in the solid state.

**Scheme 1:**
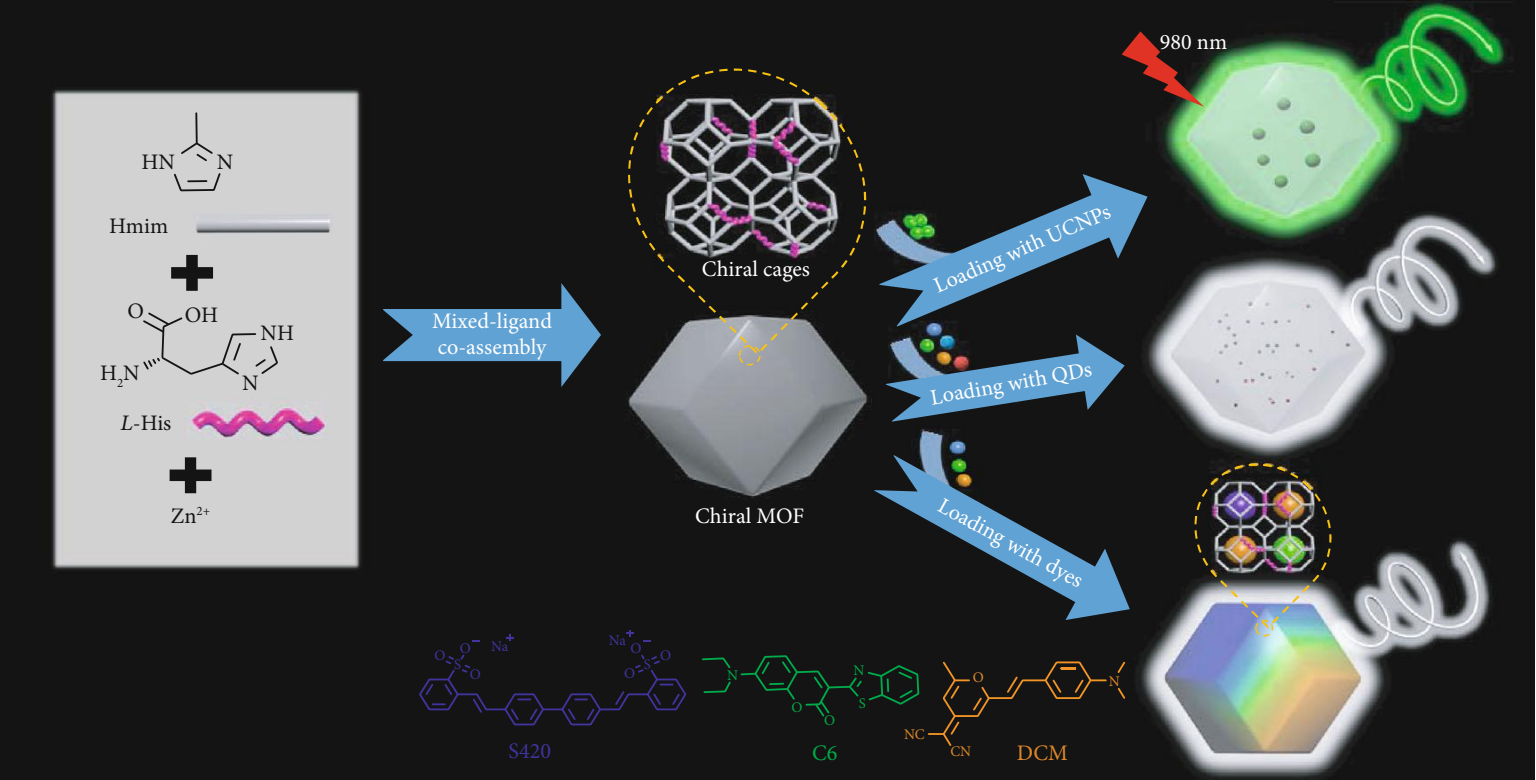
Schematic synthesis of chiral MOFs and CPL-active MOFs. Hmim and chiral histidine could coordinate with Zn ion to form chiral MOF. Various emitters, such as organic dyes, inorganic quantum dots (QDs), and lanthanide-doped upconversion nanoparticles (UCNPs), could be loaded into the chiral MOF by a simple in situ synthesis approach. The resulting emitter-loading chiral ZIFs could emit full-color circularly polarized light by tuning types of dyes or QDs, and white-color circularly polarized emission could be obtained. Additionally, upconverted circularly polarized luminescence (UC-CPL) based on achiral UCNPs and chiral MOF composites was achieved under 980 nm laser excitation.

ZIF-8 is one of the representative zeolitic imidazolate framework (a subclass of MOFs) materials with a sodalite (SOD) topology formed by Zn ions and 2-methylimidazole (Hmim) [48–51]. The crystal structure of ZIF-8 encompasses large cavities interconnected by narrow windows, which have been widely demonstrated as the host matrix for loading guest components [52, 53]. In this work, we utilized a facile and economically feasible strategy to fabricate the chiral ZIFs (*L-/D-*ZIF) by incorporating *L-/D-*histidine (His) into the ZIF-8 framework using a mixed-ligand coassembly pathway [54]. The obtained chiral ZIFs could be used as chiral host matrixes for loading various achiral emitters, such as organic dyes, inorganic QDs, and upconversion nanoparticles (UCNPs), resulting in CPL-active and upconverted CPL-active ZIFs. In addition, this approach could effectively prohibit the severe aggregation-caused emission quenching of dyes, enabling highly efficient CPL activity in the solid state. Further, the emission color of circularly polarized light could be flexibly tuned, and white circularly polarized emission could be obtained (Scheme 1). Thus, we provide a facile and general pathway for fabricating CPL-active solid-state nanomaterials through chiral ZIF host loading guest emitters.

## 2. Results

### 2.1. The Fabrication of Chiral Metal-Organic Frameworks

The chiral ZIF was synthesized by the mixed-ligand coassembly approach. For example, *L*-His and Hmim were dissolved in mixed solvent of methanol and water. Then, a small amount of triethylamine (TEA) was added into solution under stirring. After stirring for 10 minutes, the mixed-ligand solution was gradually added to the methanol solution of Zn(NO_3_)_2_·6H_2_O. Upon stirring at room temperature for 24 h, the colorless product was washed with large amounts of water and methanol, then collected by centrifugation and dried in a vacuum. First of all, powder X-ray diffraction (PXRD) was used to confirm the crystal structure of *L*-ZIF. As showed in [Supplementary-material supplementary-material-1], the PXRD patterns of *L-*ZIF were in consistence with those of ZIF-8 with identical positions, which indicated that the ZIF-8-like architecture with SOD topology formed in the framework of *L*-ZIF. However, the first-order diffraction peak of *L*-ZIF exhibited a slight shift to a larger angle which suggested a slightly smaller unit cell than that of ZIF-8 after incorporating with *L*-His ([Supplementary-material supplementary-material-1]). Meanwhile, scanning electron microscope (SEM) images of *L*-ZIF exhibited similar rhombic dodecahedral shape with ZIF-8. To confirm the successful incorporation of *L*-His into the *L*-ZIF structure, Fourier-transform infrared (FT-IR) spectroscopy and X-ray photon spectroscopy (XPS) were systematically measured. As for FT-IR spectroscopy, *L*-ZIF showed a wide peak at 1629 cm^−1^ which could ascribe to the C=O stretch in the carboxyl group from *L*-His. Additionally, vibration bands were also found around 1116 cm^−1^ and 1058 cm^−1^ in *L*-ZIF and *L*-His which were not observed in ZIF-8. XPS analysis identified the binding energy of the carboxylic group (-COOH) of *L*-ZIF at 288.75 eV from the C(1s) spectrum, while it was not presented in ZIF-8 ([Supplementary-material supplementary-material-1]). These results clearly indicated the formation of mixed-ligand chiral ZIF. To examine the chiral ligand loading efficiency, various ingredient molar ratios between *L*-His and Hmim from 1 : 7 to 1 : 2 were investigated. The final average molar ratio of these two ligands was determined by ^1^H NMR measurements (Figures [Supplementary-material supplementary-material-1] and [Supplementary-material supplementary-material-1]). The results illustrated that although different amounts of *L*-His were added, a certain molar ratio between *L*-His and Hmim is 0.1 in final products. Considering the decreased crystallization caused by the large amount of *L*-His during the synthetic process ([Supplementary-material supplementary-material-1]), the molar ratio of *n*_*L*‐His_/*n*_Hmim_ was fixed to be 1/7 in the subsequent experiments. Comprehensively considering the above results, we argue that *L*-His and Hmim are most likely randomly distributed in the extended polymeric framework skeletons of *L*-ZIF. According to the crystal structure of ZIF-8, it is clear to deduce that 1 unit cell contains 2 cages with 24 Hmim ligands, while every cage would combine 2-3 L-His as linkers ([Supplementary-material supplementary-material-1]). In addition, *D*-ZIF consisted of Zn ion, *D*-His, and Hmim showed similar properties as *L*-ZIF (Figures [Supplementary-material supplementary-material-1]). The chirality of obtained mixed-ligand ZIF was tested by circular dichroism (CD) measurement. As shown in [Supplementary-material supplementary-material-1], *L*-His exhibited a positive signal in methanol. Interestingly, a negative signal was observed in *L*-ZIF with a larger dissymmetry factor *g*_CD_ (Figures [Supplementary-material supplementary-material-1] and [Supplementary-material supplementary-material-1]). The same results were also obtained between *D*-ZIF and *D*-His. We reason that the observed reversal CD signal of the chiral ZIF should be due to the supramolecular chirality induced by the histidine unit. A similar phenomenon can be found widely in supramolecular self-assembled systems [55]. Thus, the combination of chiral ligand would endow the ZIF-8 with chirality.

### 2.2. Tunable CPL from Dye-Loaded Chiral MOFs

**Figure 1:**
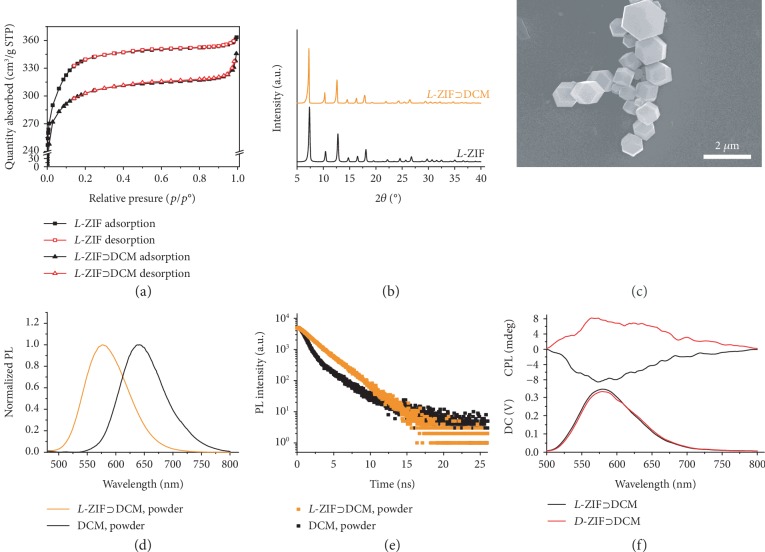
Chiral ZIF loading with DCM. (a) Nitrogen adsorption isotherm of *L*-ZIF and *L*‐ZIF⊃DCM (0.04 wt%) measured at 77 K. (b) XRD patterns of *L*-ZIF and *L*‐ZIF⊃DCM (0.04 wt%). (c) SEM image of *L*‐ZIF⊃DCM (0.04 wt%). (d) Normalized fluorescence spectra of *L*‐ZIF⊃DCM (0.04 wt%) and DCM in the solid state, *λ*_ex_ = 450 nm. (e) Fluorescence decay of *L*‐ZIF⊃DCM (0.04 wt%) and DCM in the solid state, *λ*_ex_ = 450 nm. (f) CPL spectra of *L*‐ZIF⊃DCM (0.04 wt%) and *D*‐ZIF⊃DCM (0.04 wt%) in the solid state, *λ*_ex_ = 450 nm.

Nitrogen adsorption analysis results showed that the Brunauer-Emmett-Teller (BET) surface area of *L*-ZIF was 1267.9 m^2^ g^−1^ with a micropore volume of 0.37 cm^3^ g^−1^, indicating that the *L*-ZIF is of a typical porous structure with a high surface area. By applying an *in situ* synthetic approach in which the dye molecules were simultaneously encapsulated into the framework during the synthesis of *L*-ZIF, dye-loaded chiral ZIF could be obtained. After stirring at room temperature for 24 h, the crude products were collected by centrifugation and extensively washed with large amounts of good solvent of dyes. Then, the residues were centrifuged and washed by methanol for three times followed by drying in a vacuum. From the color change or emission behavior under UV light of the ZIF powder, we could easily and quickly select appropriate guest molecules. A great number of screening tests suggested that the cationic dyes like Rhodamine B, thioflavin t (ThT), and 4-(4-dimethylaminostyryl)-1-methylpyridinium (DASPI) were difficult to be encapsulated into the cationic *L*-ZIF. On the contrary, the linear guest molecules such as anionic stilbene 420 (S 420), neutral coumarin 6 (C6), and 4-(dicyanomethylene)-2-methyl-6-(4-dimethylaminostyryl)-4H-pyran (DCM) could be loaded into *L*-ZIF easily. Therefore, the anionic or neural dyes with suitable size were required to be encapsulated into the *L*-ZIF framework. Taking DCM as an example, it was found that after loading into the *L*-ZIF, the BET surface area of *L*‐ZIF⊃DCM exhibited an obvious decrease to 1153.9 m^2^ g^−1^ (Figure 1(a)). This demonstrated that dye molecules were successfully encapsulated into the cavity of *L*-ZIF. In addition, the loading amount of DCM was 0.04 wt% determined by fluorescence spectroscopy ([Supplementary-material supplementary-material-1]). The PXRD patterns of *L*‐ZIF⊃DCM were similar to those of *L*-ZIF, which indicated that the introduction of guest dyes did not damage the crystalline structure. In addition, the slight shift to a small angle at the first-order diffraction peak demonstrated that the loading DCM enlarged the size of the unit cell due to large molecule size (Figure 1(b)). As showed in the SEM image, the morphology of *L*‐ZIF⊃DCM has no obvious change compared with that of *L*-ZIF. With or without guest DCM molecules, only uniform rhombic dodecahedron structures could be observed (Figure 1(c)). Thus, DCM molecules were effectively encapsulated into the *L*-ZIF framework interior leading to the stable and clean morphologies. The test of *L*‐ZIF⊃DCM in solid powder by confocal laser scanning microscopy (CLSM) could further confirm that DCM molecules were effectively encapsulated into the *L*-ZIF framework ([Supplementary-material supplementary-material-1]).

It was well known that DCM was a kind of aggregation-caused quenching (ACQ) dye; the photophysical properties of DCM and DCM encapsulated into *L*-ZIF in the solid state were studied. The fluorescence spectra of the *L*‐ZIF⊃DCM showed a large hypochromatic shift from 640 nm to 578 nm compared with the one of DCM powder (Figure 1(d)), which clearly indicated the good molecular distribution in *L*-ZIF cages. Furthermore, a high quantum yield (0.43) of *L*‐ZIF⊃DCM in the solid state was detected, while a poor quantum yield (0.02) was obtained in DCM powder ([Supplementary-material supplementary-material-1]). On the one hand, the molecular distribution of dye molecules in isolated *L*-ZIF cages can avoid intermolecular interactions, which will prohibit severe emission quenching. On the other hand, the good confinement of the DCM molecules within the size-matched cages of *L*-ZIF can effectively restrain the intramolecular torsional motion and increase the conformational rigidity of the dyes. Thus, the decreased ACQ effect and the improved radiative decay pathway result to the high emission efficiency of *L*‐ZIF⊃DCM. This could be further confirmed by the fluorescence lifetime testing. As showed in Figure 1(e), the fluorescence lifetime spectrum of DCM in the solid state was double-exponential decay with 1.8 ns. However, after being confined into the *L*-ZIF framework, the fluorescence decay was single-exponential mode up to 2.3 ns.

Remember that, due to the introduction of chiral histidine into the ZIF framework, the induced chirality of loaded DCM was expected. The CD spectra of *L*‐ZIF⊃DCM showed a negative signal at the absorption band of DCM, which followed the chirality of *L*-ZIF ([Supplementary-material supplementary-material-1]). In addition, DCM-loaded *D*-ZIF was also successfully achieved using the same way ([Supplementary-material supplementary-material-1]). Interestingly, *D*‐ZIF⊃DCM exhibited a positive CD signal, which was opposite to *L*‐ZIF⊃DCM. This indicated the authentic induced chirality from chiral confined space to achiral dyes. The guest dye DCM-loaded *L*-/*D*-ZIF showed significant fluorescence enhancement which encourages us to investigate the CPL activity. Amazingly, strong CPL signals were detected from these host-guest systems with a completely opposite curve (Figure 1(f)), while no obvious signal could be observed in ZIF‐8⊃DCM ([Supplementary-material supplementary-material-1]). The magnitude of CPL can be evaluated according to the dissymmetric factor (*g*_lum_), which is defined as *g*_lum_ = 2 × (*I*_L_‐*I*_R_)/(*I*_L_ + *I*_R_), in which *I*_L_ and *I*_R_ represent the intensity of left- and right-handed circularly polarized light, respectively. The maximum *g*_lum_ value ranges from +2 to -2. The calculated value of *g*_lum_ from *L*‐/*D*‐ZIF⊃DCM is about ±1.2 × 10^−3^, which is a comparable value for organic small molecules. It must be mentioned that the induced chirality of DCM could be regulated by the chirality of *L*-/*D*-ZIF matrixes. These results revealed that the initial chirality of chiral matrixes played a vital role for the chiral transfer.

**Figure 2:**
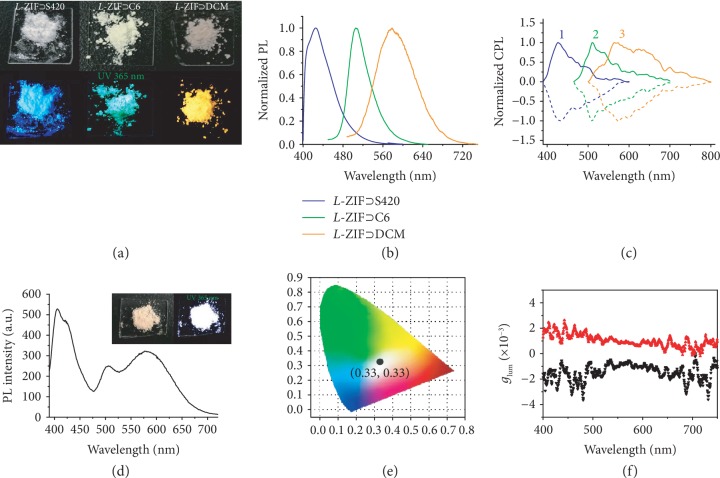
Tunable CPL form chiral ZIF⊃dyes. (a) Photographs of *L*‐ZIF⊃S420 (0.3 wt%), *L*‐ZIF⊃C6 (0.04 wt%), and *L*‐ZIF⊃DCM (0.04 wt%) under light (top) and UV light (bottom, 365 nm). (b) Normalized fluorescence spectra of *L*‐ZIF⊃S420 (0.3 wt%), *L*‐ZIF⊃C6 (0.04 wt%), and *L*‐ZIF⊃DCM (0.04 wt%), excitation by 450 nm but 360 nm for *L*‐ZIF⊃S420. (c) Mirror-image CPL spectra of (1) *L*‐/*D*‐ZIF⊃S420 (0.3 wt%), (2) *L*‐/*D*‐ZIF⊃C6 (0.04 wt%), and (3) *L*‐/*D*‐ZIF⊃DCM (0.04 wt%), excitation by 450 nm but 360 nm for *L*‐/*D*‐ZIF⊃S420. All the dashed lines are the CPL signal obtained from *L*‐ZIF⊃dye; all the solid lines are the CPL signal obtained from *D*‐ZIF⊃dye. (d) Fluorescence spectra of the white light-emitting *L*‐ZIF⊃S420/C6/DCM (0.02 wt% S420, 0.03 wt% C6, and 0.03 wt% DCM) with excitation wavelength of 370 nm. Inset: photograph of the *L*‐ZIF⊃S420/C6/DCM (0.02 wt% S420, 0.03 wt% C6, and 0.03 wt% DCM) under light (left) and UV light (right, 365 nm). (e) The CIE coordinate of *L*‐ZIF⊃S420/C6/DCM (0.02 wt% S420, 0.03 wt% C6, and 0.03 wt% DCM). (f) CPL dissymmetric factor *g*_lum_ as a function of the wavelength (black: *L*‐ZIF⊃S420/C6/DCM; red: *D*‐ZIF⊃S420/C6/DCM), *λ*_ex_ = 360 nm.

Encouraged by these results, the encapsulation of the blue- and green-emitting guest molecules into *L*-ZIF was further investigated. Fluorescent brightener (S420) and the coumarin derivative (C6) in methanol solution display intense blue and green emission bands centered at about 423 nm and 508 nm, respectively ([Supplementary-material supplementary-material-1]). Fortunately, they could also be easily loaded into the cavities of *L-*/*D*-ZIF, while the emission properties were similar to the above-demonstrated *L*‐/*D*‐ZIF⊃DCM (Figures [Supplementary-material supplementary-material-1]). Considering that the anionic S420 may coordinate with Zn ion during the synthetic process, we have carefully investigated the additive amount of S420 in the reaction mixtures. It was found that high dye concentration (over 2 mM) not only affected the chirality of final *L*‐ZIF⊃S420 but also led to the damage of morphology as well as crystallinity of *L*‐ZIF⊃S420 (Figures [Supplementary-material supplementary-material-1] and [Supplementary-material supplementary-material-1]). Thus, after optimizing the usage of S420 in the fabrication process, we fixed the concentration to 1 mM. Under UV irradiation, the powder of *L*‐ZIF⊃S420, *L*‐ZIF⊃C6, and *L*‐ZIF⊃DCM exhibited bright blue, green, and orange emission, respectively (Figures 2(a) and 2(b)). Furthermore, the fluorescence spectrum of *L*‐ZIF⊃S420 showed blue emission bands centered at 426 nm with enhanced emission efficiency from 37% to 59% compared with S420 powder ([Supplementary-material supplementary-material-1]). A similar phenomenon was observed from chiral ZIF⊃C6, and the highest emission enhancement was observed in *D*‐ZIF⊃C6 with quantum yield 76%. These further indicated that the guest dyes were homogeneously distributed inside the *L*-ZIF framework without severe aggregation. Additionally, induced CPL could be observed in all the tested chiral ZIF⊃dyes and were found to exhibited mirror-image signal from 426 nm to 578 nm (Figure 2(c)). The ∣*g*_lum_∣ values obtained from chiral ZIF-encapsulated S420 and C6 were 9.0 × 10^−4^ and 3.0 × 10^−4^, respectively. In addition, the Commission International de l'Éclairage (CIE) coordinates calculated from *L*‐ZIF⊃S420, *L*‐ZIF⊃C6, and *L*‐ZIF⊃DCM were (0.15, 0.07), (0.19, 0.59), and (0.49, 0.50), respectively ([Supplementary-material supplementary-material-1]), which covered the blue via green to orange circularly polarized emission. To date, this kind of full-color covering CPL systems was rarely reported in the solid state especially in these chirality-induced host-guest systems.

Materials with white light emission are particularly important, and plenty of studies on achieving white emission MOFs have been reported [38, 56, 57]. However, to date, MOFs with white-color circularly polarized light emission have never been reported. Based on the previous results, it was found that *L*‐ZIF⊃S420, *L*‐ZIF⊃C6, and *L*‐ZIF⊃DCM showed blue, green, and orange emission, respectively. Encouraged by these results, if S420, C6, and DCM are encapsulated into *L*-ZIF compatibly, white light can be realized through balancing the contents of their emissions. Encouragingly, the *L*-ZIF composite, *L*‐ZIF⊃S420/C6/DCM, was obtained simply by adding mixed solution of S420, C6, and DCM during the synthesis process. According to the screening tests, white light-emitting *L*‐ZIF⊃S420/C6/DCM was achieved under excitation at 370 nm (Figure 2(d)). The fluorescence spectrum of *L*‐ZIF⊃S420/C6/DCM covered almost the whole visible spectral region, with the CIE chromaticity coordinates measured as (0.33, 0.33), the same as that of the ideal white light (Figure 2(e)). Furthermore, the white light is kept when varying the excitation wavelength from 335 nm to 380 nm ([Supplementary-material supplementary-material-1]). Most importantly, for the first time, we succeeded in achieving circularly polarized white light emission in a luminescent MOF system ([Supplementary-material supplementary-material-1]). A reasonably large spectral width of 400 to 750 nm was observed in the CPL spectra. After converting to *g*_lum_ spectra, nearly parallel diagrams were obtained (Figure 2(f)). The luminescence dissymmetry factor ∣*g*_lum_∣ was round 1 × 10^−3^, which was the same as those of the individual chiral ZIF⊃dye. This method thus provides a simple and general approach for fabricating luminescent MOFs that emit white circularly polarized light.

### 2.3. Tunable CPL from QD-Loaded Chiral MOFs

**Figure 3:**
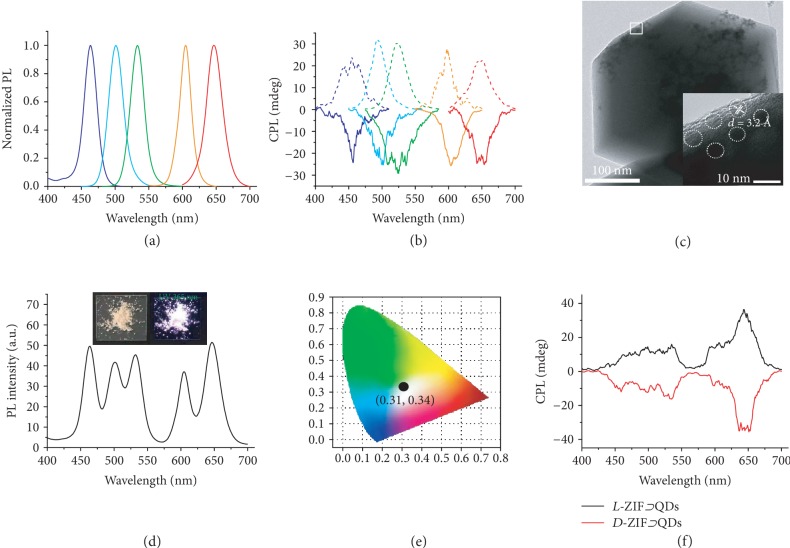
Tunable CPL form chiral ZIF⊃QDs. (a) Normalized fluorescence spectra of corresponding *L*‐ZIF⊃QD composites in the solid state, *λ*_ex_ = 360 nm. (b) Mirror-image CPL spectra of corresponding *L*‐ZIF⊃QD composites in the solid state, *λ*_ex_ = 360 nm. All the dashed lines are the CPL signal obtained from *L*‐ZIF⊃QD; all the solid lines are the CPL signal obtained from *D*‐ZIF⊃QD. (c) TEM and HRTEM images of *L*‐ZIF⊃QD533. (d) Fluorescence spectra of white light-emitting *L*‐ZIF⊃QDs in the solid state, *λ*_ex_ = 360 nm. Inset: photograph of the *L*‐ZIF⊃QDs under light (left) and UV light (right, 365 nm). (e) The CIE coordinates of *L*‐ZIF⊃QDs, *λ*_ex_ = 360 nm. (f) CPL spectra of *L*‐ZIF⊃QDs and *D*‐ZIF⊃QDs in the solid state, *λ*_ex_ = 360 nm.

Besides organic dyes, the incorporation of semiconductor QDs in chiral ZIFs has been also achieved in this work. For example, during the preparation of *L*‐ZIF⊃QD533 (with green-color emission) composites, the functionalization of the QD surface with polyvinylpyrrolidone (PVP) was firstly carried on. Then, *L*‐ZIF⊃QD533 composites were prepared by the crystallization of *L*-ZIF in methanol at room temperature in the presence of PVP-modified QD533. The optimizing concentration of QD533 during the synthesis process was 0.3 mg/mL ([Supplementary-material supplementary-material-1]). PXRD indicated that the crystal structure of *L*-ZIF remained after the incorporation with QDs. Although the CD signal of *L*‐ZIF⊃QD533 was not detected due to the strong scattering, the intense CPL signal of *L*‐ZIF⊃QD533 can be observed at the corresponding emission region ([Supplementary-material supplementary-material-1]). Interestingly, the CPL of QD533 in *L*-ZIF exhibited a positive signal with *g*_lum_ value 4.6 × 10^−3^ at 533 nm, an opposite and relatively larger value than that of *L*‐ZIF⊃dye ([Supplementary-material supplementary-material-1]). Considering that the size of QD533 was much larger than the cage size of *L*-ZIF, QDs were beyond the cages and dispersed into the crystals of *L*-ZIF. Therefore, the inverted CPL signals resulted from the induction outside of the surrounding cages. Otherwise, *D*‐ZIF⊃QD533 composites were fabricated, and a negative sign of CPL was detected with the same order of magnitudes of *g*_lum_ value −4.3 × 10^−3^ as *L*‐ZIF⊃QD533. This indicated that the induced chirality of QDs could be controlled by the inherent chirality of chiral ZIFs. In addition, the wavelength of CPL could be tuned by simply changing the type of QDs (Figures [Supplementary-material supplementary-material-1] and [Supplementary-material supplementary-material-1]). As shown in Figures 3(a) and 3(b) and S29b, the color of CPL from chiral ZIF⊃QD composites could cover the range from blue *via* cyan, green, and orange to red in the solid state. To date, this kind of full-color covering CPL-active systems from semiconductor QDs in the solid state has rarely been reported due to the tedious synthesis. To further confirm the origin of chirality of QDs, transmission electron microscopy (TEM) imaging of ZIF⊃QD533 was performed (Figure 3(c) and [Supplementary-material supplementary-material-1]). The TEM images showed that QD533 was encapsulated inside the *L*-ZIF particles while the high-resolution TEM (HRTEM) image indicated that the original crystal structure of QD533 was preserved. These results illustrated that the detected CPL signal of chiral ZIF⊃QD533 resulted from the induced chirality by confined chiral environment from chiral ZIFs [58, 59]. In addition, through tuning the mass ratio of five kinds of QDs loaded in chiral ZIF, chiral ZIF⊃QDs with white circularly polarized light emission also could be obtained in the solid state. As showed in Figures 3(d)–3(f) and [Supplementary-material supplementary-material-1], white circularly polarized light-emitting chiral ZIFs showed mirror-image CPL signal from 400 nm to 700 nm with a ∣*g*_lum_∣ value around 5.0 × 10^−3^.

### 2.4. Upconverted CPL from UCNP-Loaded Chiral MOFs

**Figure 4:**
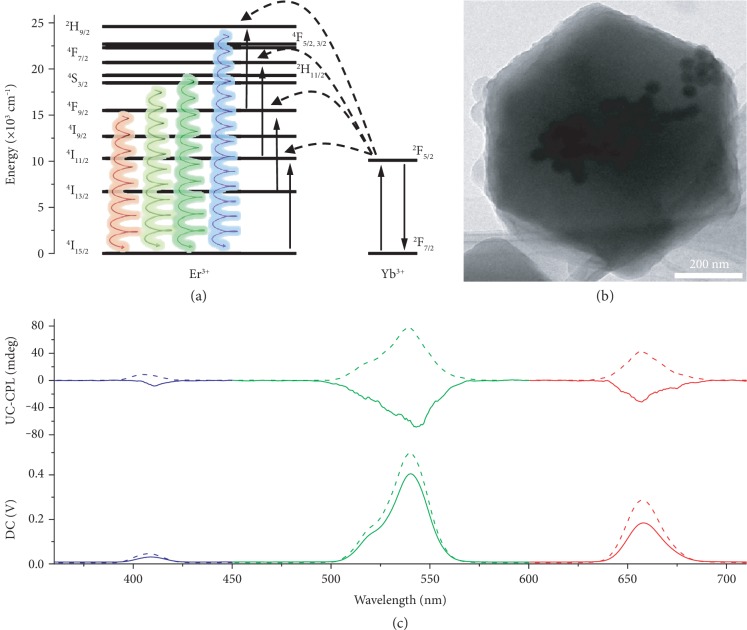
UC-CPL from chiral ZIF⊃UCNP. (a) Simplified energy level diagram and energy-transfer upconversion process for the NaYF_4_:Yb, Er UCNPs. The spirals represent the detected UC-CPL in this work. (b) TEM image of *L*‐ZIF⊃UCNP : Er. (c) CPL spectra of *L*‐ZIF⊃UCNP : Er (dashed line) and *D*‐ZIF⊃UCNP : Er (solid line), *λ*_ex_ = 980 nm laser.

Encouraged by the results from QDs, one type of lanthanide-loaded upconversion nanoparticles (UCNPs)—NaYF_4_:Yb, Er—was also encapsulated into the chiral ZIFs by a similar strategy. Excitingly, the upconverted CPL (UC-CPL) could be observed under the excitation of 980 nm laser (Figure 4(a)). Firstly, the photoluminescence of UCNP:Er was not changed after loading into *L*-ZIF, and the crystallinity of *L*-ZIF was reserved ([Supplementary-material supplementary-material-1]). Additionally, the upconverted emission intensity of *L*‐ZIF⊃UCNP : Er was found to be closely dependent on the excitation power density with the slope of 2.0 at 541 nm, which indicated a two-photon absorption-based upconversion ([Supplementary-material supplementary-material-1]). As showed in Figure 4(c), the mirror-image UC-CPL could be observed from 390 nm (navy) to 700 nm (red). More intriguingly, due to the relatively large *g*_lum_ value (~ ± 1.2 × 10^−2^) of UC-CPL [12], even some emission peaks showed lower intensity under the same excitation, a distinct CPL signal could be detected (Figure 4(c) and [Supplementary-material supplementary-material-1]). Similar to the QD-loaded ZIF, TEM images showed that UCNP:Er was distributed inside the ZIF particles (Figures 4(b) and [Supplementary-material supplementary-material-1]), which indicated that the induced UC-CPL was resulting from the chiral confinement effect of chiral ZIFs.

## 3. Discussion

**Figure 5:**
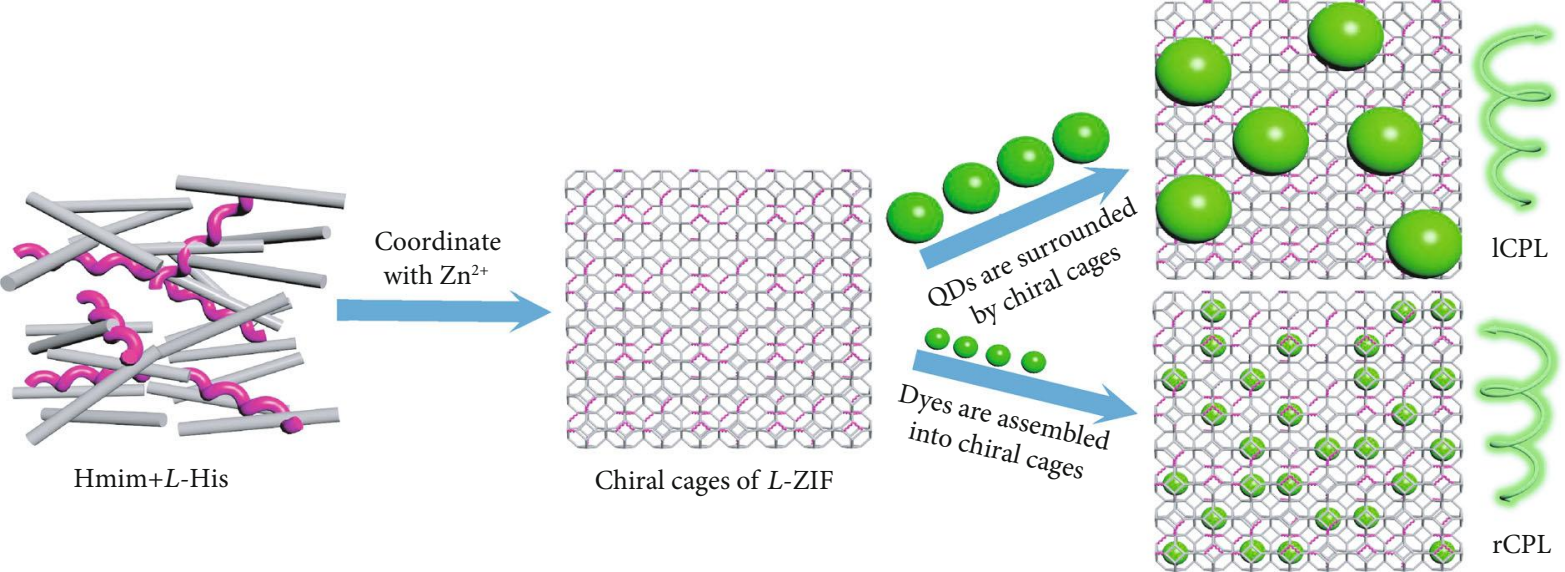
Schematic illustration of induced CPL with opposite polarization from dyes and QDs in chiral MOF. Well-arranged chiral cages are formed by the mixed ligands through coordination with Zn ions (gray rods: Hmim, purple spirals: *L*-His). The size-matched dyes assemble into the chiral cages during framework formation. Thus, the induced CPL of dyes follows the chirality of cages with right-handed polarization (rCPL). However, the large-sized QDs will distribute into the framework. It is surrounded by the chiral cages, which show inverted CPL with left-handed polarization (lCPL).

It has been demonstrated that the chiral confined space or environment can endow achiral components with chirality [17, 29, 60]. The induced chirality is caused by dipole-dipole interaction between guest molecules and chiral hosts [61, 62]. In this work, the incorporation of *L*-/*D*-His in ZIF crystals makes the ZIFs with chirality, which can endow chirality to the encapsulated guest emitters. Interestingly, the loaded dyes and QDs exhibit opposite CPL signals in homochiral ZIFs. Considering that the cage size of ZIF-8 is 11.6 Å [48] while the diameter of QDs is approximately 6 nm ([Supplementary-material supplementary-material-1]), it should be noted that the small dyes can assemble into the cages of chiral ZIFs while QDs are too large to be encapsulated into these chiral cages. Based on these results, a mechanism of dual package mode-inducted CPL is proposed (Figure 5). Through the *in situ* synthesis process, the size-matched dyes are assembled into the chiral cages. Thus, the induced CPL of dyes followed the chirality of ZIFs with clockwise polarization. However, due to the large size, the QDs are at the outside of the cages, *i.e.*, directly distributed into the framework and surrounded by the chiral ZIF. In this case, the induction of CPL, showing anticlockwise polarization, originates from the outside of chiral cages with inverted chirality [63, 64]. Thus, the dual encapsulation mode-induced CPL are observed from chiral ZIFs (Figure 5).

In summary, we have achieved solid-state CPL-active materials with high luminescence efficiency by introducing achiral dyes, QDs, and UCNPs into chiral MOF matrixes. The emitting colors can be flexibly tuned by changing the categories of dyes and QDs. In addition, white light CPL-emitting MOF materials are obtained by introducing various dyes or QDs simultaneously. Additionally, the chiral MOFs resulting from the mixed-ligand coassembly provided different encapsulation modes for dyes and QDs. Dyes are encapsulated in chiral cages while QDs are distributed in the gaps of the framework, resulting in remarkable CPL activity. Meanwhile, UC-CPL with a high dissymmetric factor is achieved through loading UCNPs into the chiral MOF matrixes. As there are so many chiral porous MOFs and various luminophores, lots of applications, such as circularly polarized white light-emitting diodes (CPWLEDs), circularly polarized laser, and upconverted asymmetric photocatalysis, remind us to continue exploring this field.

## 4. Materials and Methods

### 4.1. Materials

All reagents and solvents were used as received, unless otherwise indicated. Zinc nitrate hexahydrate and 2-methylimidazole were purchased from Sigma-Aldrich. Polymethylpyrrolidone (PVP, Mw = 10000), L-histidine, and D-histidine were purchased from TCI (Shanghai) Development Co., Ltd. YbCl_3_·6H_2_O (99.9%), YCl_3_·6H_2_O (99.99%), and ErCl_3_ (99.9%) were purchased from Innochem (Beijing) Co., Ltd. All the used quantum dots were purchased from Suzhou Xingshuo Nanotech Co., Ltd.

### 4.2. Characterization

The ^1^H NMR spectra were recorded on a Bruker Fourier 400 (400 MHz) spectrometer. X-ray diffraction (XRD) was achieved on a Rigaku D/Max-2500 X-ray diffractometer (Japan) with Cu/K*α* radiation (*λ* = 1.5406 Å), and XPS was measured on an ESCALAB250XI spectrometer. Nitrogen adsorption isotherm was tested on a ASAP2420-4 instrument at 77 K. Transmission electron microscopy (TEM) and high-resolution transmission electron microscopy (HRTEM) images were taken on a Tecnai G2 F20 U-TWIN microscope (200 kV); before testing, the samples were suspended on carbon-coated Cu grids. Scanning electron microscopy (SEM) was performed on a Hitachi S-4800 FE-SEM with an accelerating voltage of 10 kV. Before SEM proceeded, the samples on silicon wafers were coated with a thin layer of Pt to increase the contrast. FT-IR spectra were collected on the S-ONE spectrometer utilizing an HATR mode. UV-vis spectra were obtained using a Hitachi U-3900 spectrophotometer, and fluorescence spectra were measured on a Zolix Omni-*λ*500i monochromator with photomultiplier tube PMTH-R 928 using a Xe lamp as the excitation source. CD spectra were measured on a JASCO J-815 spectrophotometer. CPL spectra were obtained using a JASCO CPL-200 spectrophotometer. Confocal laser scanning microscopy was recorded on the Olympus FV1000-IX81. The absolute fluorescence quantum yield was measured by using an absolute PL quantum yield spectrometer (Edinburg FLS-980 fluorescence spectrometer) with a calibrated integrating sphere, and fluorescence lifetime measurements were recorded on a NanoLog spectrometer using e chiral confinement effect of chiral.

Further details of the materials and methods are included in Supplementary Materials ([Supplementary-material supplementary-material-1]).

## Data Availability

All data needed to evaluate the conclusions in the paper are present in the paper and Supplementary Materials. Additional data related to this paper may be requested from the authors.

## Supplementary Materials

Supplementary Materials Figure S1: PXRD patterns and SEM images of L-ZIF and ZIF-8. Figure S2: FT-IR, XPS, and ^1^H NMR spectra of L-ZIF and ZIF-8. Figure S3: ^1^H NMR spectra of L-ZIF. Figure S4: PXRD patterns of L-ZIF and ZIF-8. Figure S5: schematic representation of the synthetic process of chiral cages. Figure S6: PXRD patterns of D-ZIF and ZIF-8. Figure S7: SEM image, XPS, and FT-IR spectra of D-ZIF and ZIF-8. Figure S8: ^1^H NMR spectra of D-ZIF. Figure S9: chiral amplification and inversion between L-/D-His and L-/D-ZIF. Figure S10: LSCM image of L‐ZIF⊃DCM. Figure S11: induced chirality of L‐/D‐ZIF⊃DCM. Figure S12: PXRD patterns and SEM images of D‐ZIF⊃DCM. Figure S13: fluorescence of S420 and C6 in methanol solution. Figure S14: PXRD patterns and SEM images of L‐ZIF⊃S420. Figure S15: PXRD patterns and SEM images of D‐ZIF⊃S420. Figure S16: the concentration of S420 influenced CPL. Figure S17: the concentration of S420 influenced morphology. Figure S18: induced chirality of L‐/D‐ZIF⊃S420. Figure S19: PXRD patterns and SEM images of L‐ZIF⊃C6. Figure S20: PXRD patterns and SEM images of D‐ZIF⊃C6. Figure S21: induced chirality of L‐/D‐ZIF⊃C6. Figure S22: LSCM images of L‐ZIF⊃S420 and L‐ZIF⊃C6. Figure S23: CIE coordinates of luminescent MOFs. Table S1: photophysical parameters of dyes and L‐/D‐ZIF⊃dyes in solid state. Figure S24: white light-emitting MOFs with CPL. Figure S25: loading efficiency of S420. Figure S26: loading efficiency of C6. Figure S27: loading efficiency of DCM. Figure S28: induced chirality of L‐/D‐ZIF⊃QD533. Figure S29: fluorescence of QDs. Figure S30: QD-loaded chiral MOFs. Table S2: dissymmetry factor of CPL-active MOFs. Figure S31: white light-emitting MOFs with CPL. Figure S32: UCNP-loaded L-ZIF. Figure S32: photon upconverted chiral MOFs. Figure S34: UCNP-loaded D-ZIF. Figure S35: TEM image of QDs. Table S3: dissymmetry factor of upconverted CPL-active MOFs.
