# Supplementary material for: Dual-Mode Induction of Tunable Circularly Polarized Luminescence from Chiral Metal-Organic Frameworks
Source: Research (Wash D C). 2020 Jan 23;2020:6452123. doi: 10.34133/2020/6452123 (PMC6998039; doi:10.34133/2020/6452123)
Supplement: Supplementary Materials — Figure S1: PXRD patterns and SEM images of L-ZIF and ZIF-8. Figure S2: FT-IR, XPS, and 1H NMR spectra of L-ZIF and ZIF-8. Figure S3: 1H NMR spectra of L-ZIF. Figure S4: PXRD patterns of L-ZIF and ZIF-8. Figure S5: schematic representation of the synthetic process of chiral cages. Figure S6: PXRD patterns of D-ZIF and ZIF-8. Figure S7: SEM image, XPS, and FT-IR spectra of D-ZIF and ZIF-8. Figure S8: 1H NMR spectra of D-ZIF. Figure S9: chiral amplification and inversion between L-/D-His and L-/D-ZIF. Figure S10: LSCM image of L‐ZIF⊃DCM. Figure S11: induced chirality of L‐/D‐ZIF⊃DCM. Figure S12: PXRD patterns and SEM images of D‐ZIF⊃DCM. Figure S13: fluorescence of S420 and C6 in methanol solution. Figure S14: PXRD patterns and SEM images of L‐ZIF⊃S420. Figure S15: PXRD patterns and SEM images of D‐ZIF⊃S420. Figure S16: the concentration of S420 influenced CPL. Figure S17: the concentration of S420 influenced morphology. Figure S18: induced chirality of L‐/D‐ZIF⊃S420. Figure S19: PXRD patterns and SEM images of L‐ZIF⊃C6. Figure S20: PXRD patterns and SEM images of D‐ZIF⊃C6. Figure S21: induced chirality of L‐/D‐ZIF⊃C6. Figure S22: LSCM images of L‐ZIF⊃S420 and L‐ZIF⊃C6. Figure S23: CIE coordinates of luminescent MOFs. Table S1: photophysical parameters of dyes and L‐/D‐ZIF⊃dyes in solid state. Figure S24: white light-emitting MOFs with CPL. Figure S25: loading efficiency of S420. Figure S26: loading efficiency of C6. Figure S27: loading efficiency of DCM. Figure S28: induced chirality of L‐/D‐ZIF⊃QD533. Figure S29: fluorescence of QDs. Figure S30: QD-loaded chiral MOFs. Table S2: dissymmetry factor of CPL-active MOFs. Figure S31: white light-emitting MOFs with CPL. Figure S32: UCNP-loaded L-ZIF. Figure S32: photon upconverted chiral MOFs. Figure S34: UCNP-loaded D-ZIF. Figure S35: TEM image of QDs. Table S3: dissymmetry factor of upconverted CPL-active MOFs. [file 6452123.f1.docx]

Supporting Information

**Dual-Mode Induction of Tunable Circularly Polarized Luminescence from Chiral Metal-organic Frameworks**

Tonghan Zhao^1,4^, Jianlei Han^1^, Xue Jin^1^, Minghao Zhou^1^, Yan Liu^3^, Pengfei Duan*^1,4^ and Minghua Liu*^1,2,4^

^1^CAS Center for Excellence in Nanoscience, CAS Key Laboratory of Nanosystem and Hierarchical Fabrication, National Center for Nanoscience and Technology (NCNST), No. 11 ZhongGuanCun BeiYiTiao, 100190 Beijing, P.R. China.

^2^Beijing National Laboratory for Molecular Science, CAS Key Laboratory of Colloid, Interface and Chemical Thermodynamics, Institute of Chemistry, Chinese Academy of Sciences, No.2, ZhongGuanCun BeiYiJie, Beijing 100190, P. R. China.

^3^School of Chemistry and Chemical Engineering, Shanghai Jiao Tong University, Shanghai 200240, P. R. China.

^4^University of Chinese Academy of Sciences, Beijing 100049, P. R. China.

*Correspondence should be addressed to Pengfei Duan: duanpf@nanoctr.cn, and Minghua Liu: liumh@iccas.ac.cn

**S1. Synthesis and characterization of chiral ZIFs**

(1) Synthesis of ZIF-8:

Methanol solution (15 mL) of 2-methylimidazole (300 mg, 3.6 mmol) was gradually added to the methanol solution (15 mL) of Zn(NO_3_)_2_•6H_2_O (270 mg, 0.9 mmol). The reaction was carried out stirring at room temperature for 24h. The resulting product was collected by centrifugation and repeatedly washed with 30 mL methanol four times. The collected colorless powder was dried in vacuum.

(2) Synthesis of *L*-ZIF:

A mixture of 2-methylimidazole (260 mg, 3.15 mmol) and L-histidine (70 mg, 0.45 mmol) was dissolved in 15 mL mixed solution of H_2_O/methanol (2:3 v/v) equipped with a magnetic stirring bar. Then 60 μL triethylamine was added followed by stirring for 10 min. After that, the mixed-ligand solution was gradually added to the methanol solution (15 mL) of Zn(NO_3_)_2_•6H_2_O (270 mg, 0.9 mmol). The reaction was carried out stirring at room temperature for 24h. The resulting product was collected by centrifugation and repeatedly washed with 30 mL H_2_O/methanol (2:3 v/v) four times. The collected colorless powder was dried in vacuum.

D-ZIF was synthesized as same as L-ZIF excepted D-histidine was instead of L-histidine.

**Determination of histidine contents.** Well-dried histidine incorporated ZIF powder (~ 5 mg) was redispersed in 5 mL methanol, then moderate diluted hydrochloric acid was added to decompose ZIF powder. After removed solvent and hydrochloric acid, the contents of histidine was studied by ^1^H-NMR.


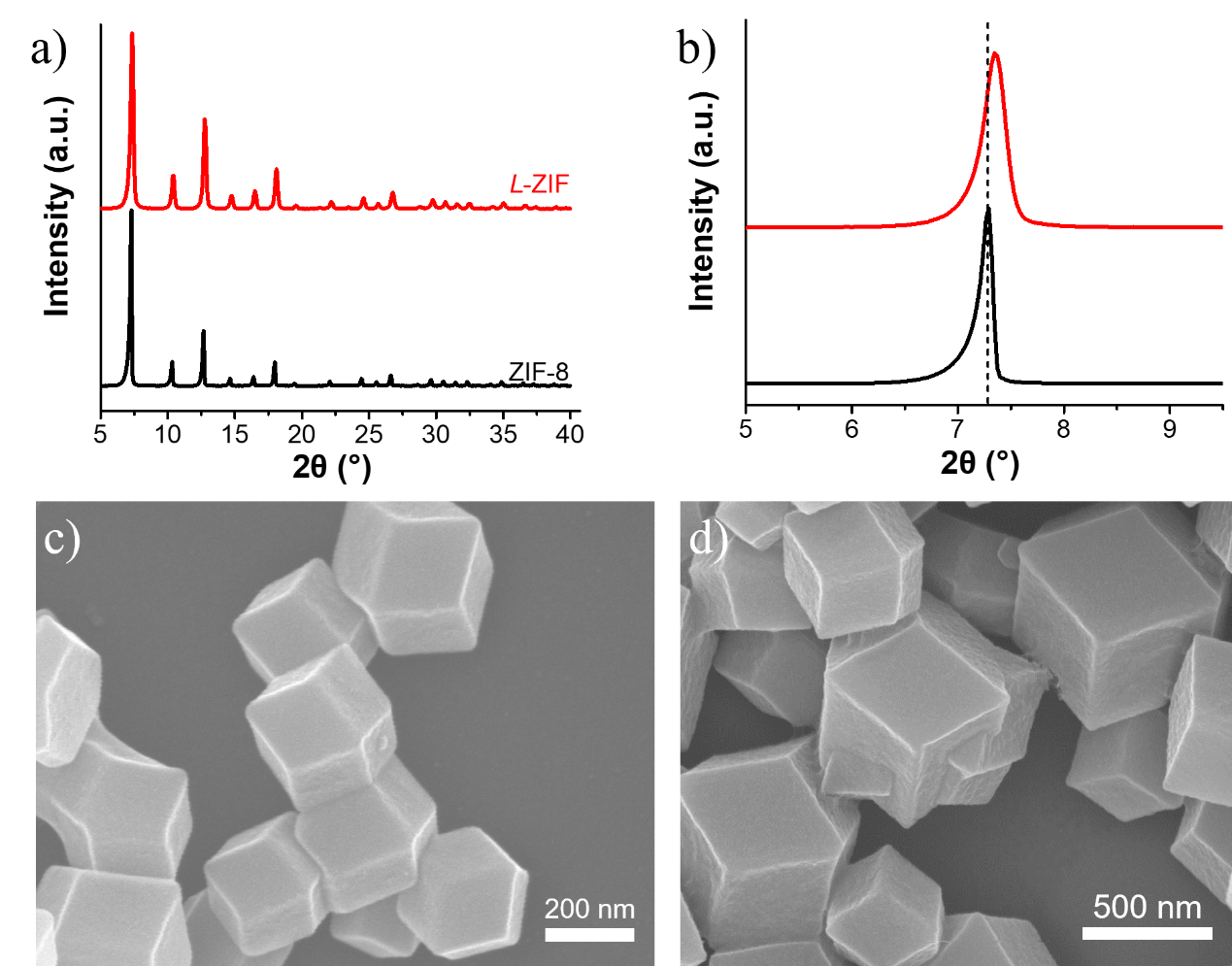


**Figure S1.** a) PXRD patterns of L-ZIF and ZIF-8. b) Enlarged PXRD patterns of L-ZIF and ZIF-8 at first-order diffraction peak. SEM images of c) ZIF-8 and d) L-ZIF.


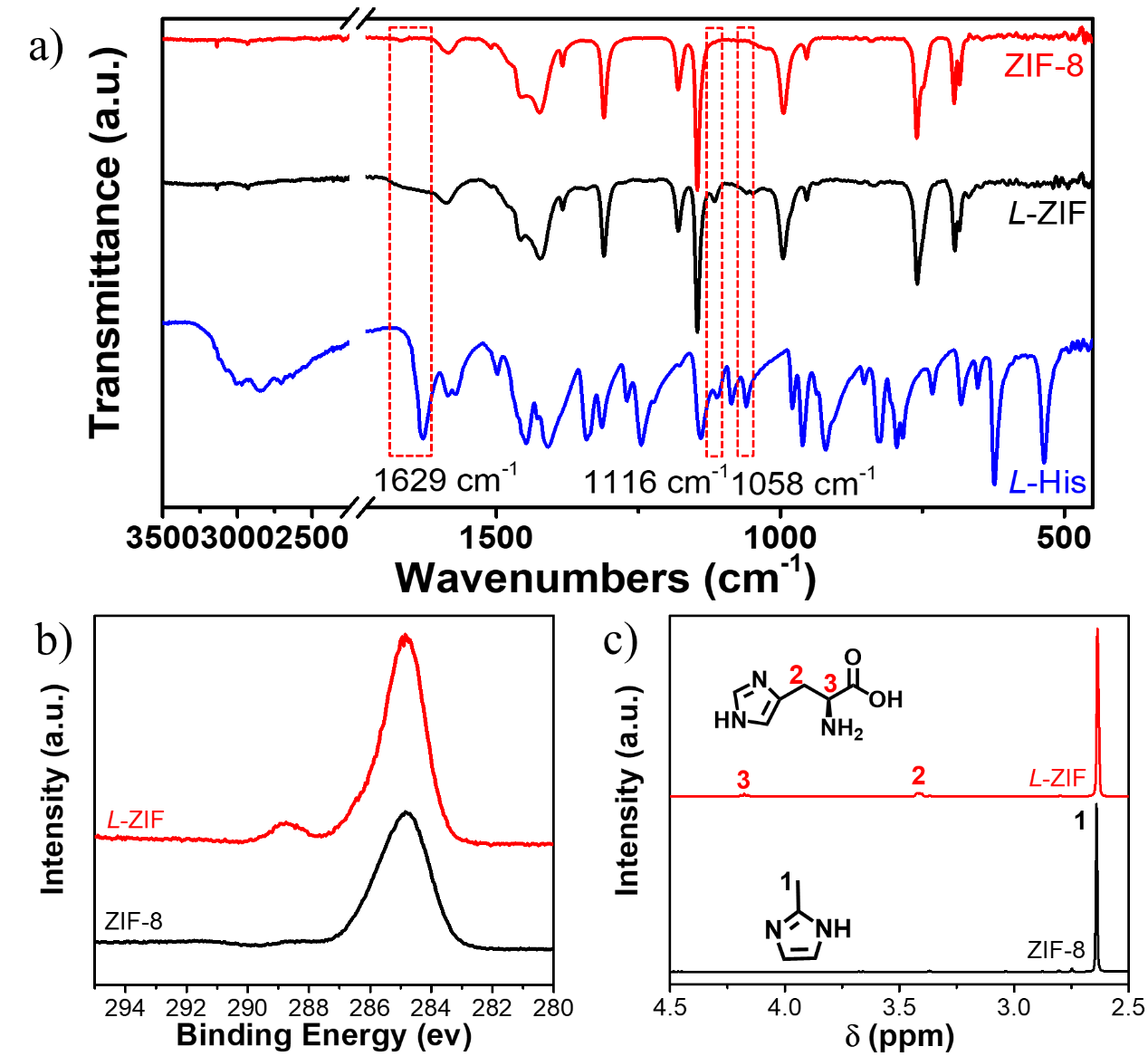


**Figure S2.** a) FT-IR spectra of L-ZIF, L-His and ZIF-8. b) XPS analysis of C1s between L-ZIF and ZIF-8 crystals. c) Solution ^1^H NMR spectra of acid-digested ZIF-8 and L-ZIF in D_2_O.


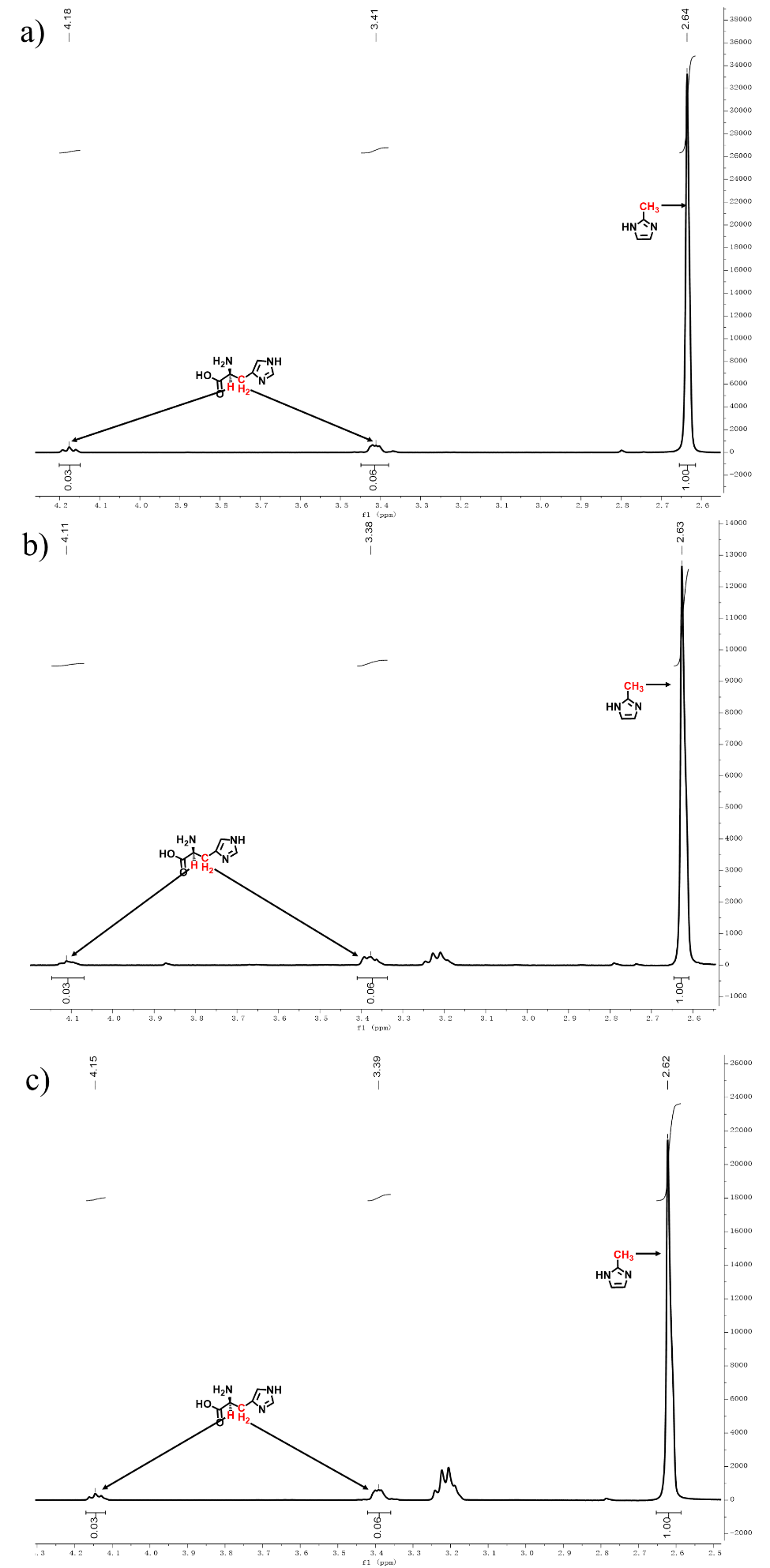


**Figure S3.** a) Solution ^1^H-NMR of acid-digested L-ZIF obtained from n_Hmim_/n_His_ = 7/1 during the synthesized process. b) Solution ^1^H-NMR of acid-digested L-ZIF obtained from n_Hmim_/n_His_ = 4/1 during the synthesized process. c) Solution ^1^H-NMR of acid-digested L-ZIF obtained from n_Hmim_/n_His_ = 2/1 during the synthesized process.


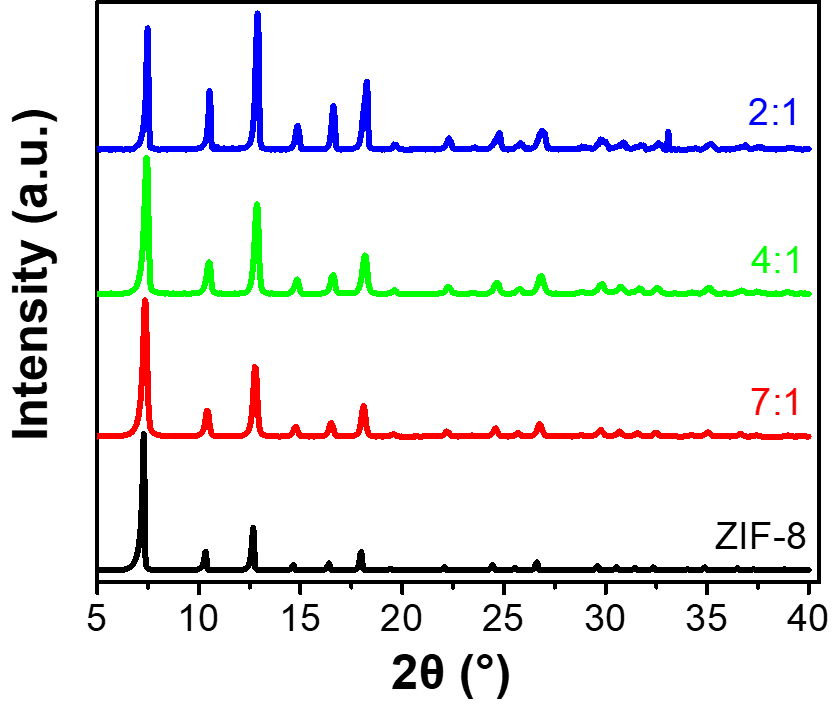


**Figure S4.** XRD patterns of ZIF-8 (black line) and L-ZIF (red line, n_Hmim_/n_His_ = 7/1 during the synthesized process; green line, n_Hmim_/n_His_ = 4/1 during the synthesized process; blue line, n_Hmim_/n_His_ = 2/1 during the synthesized process).


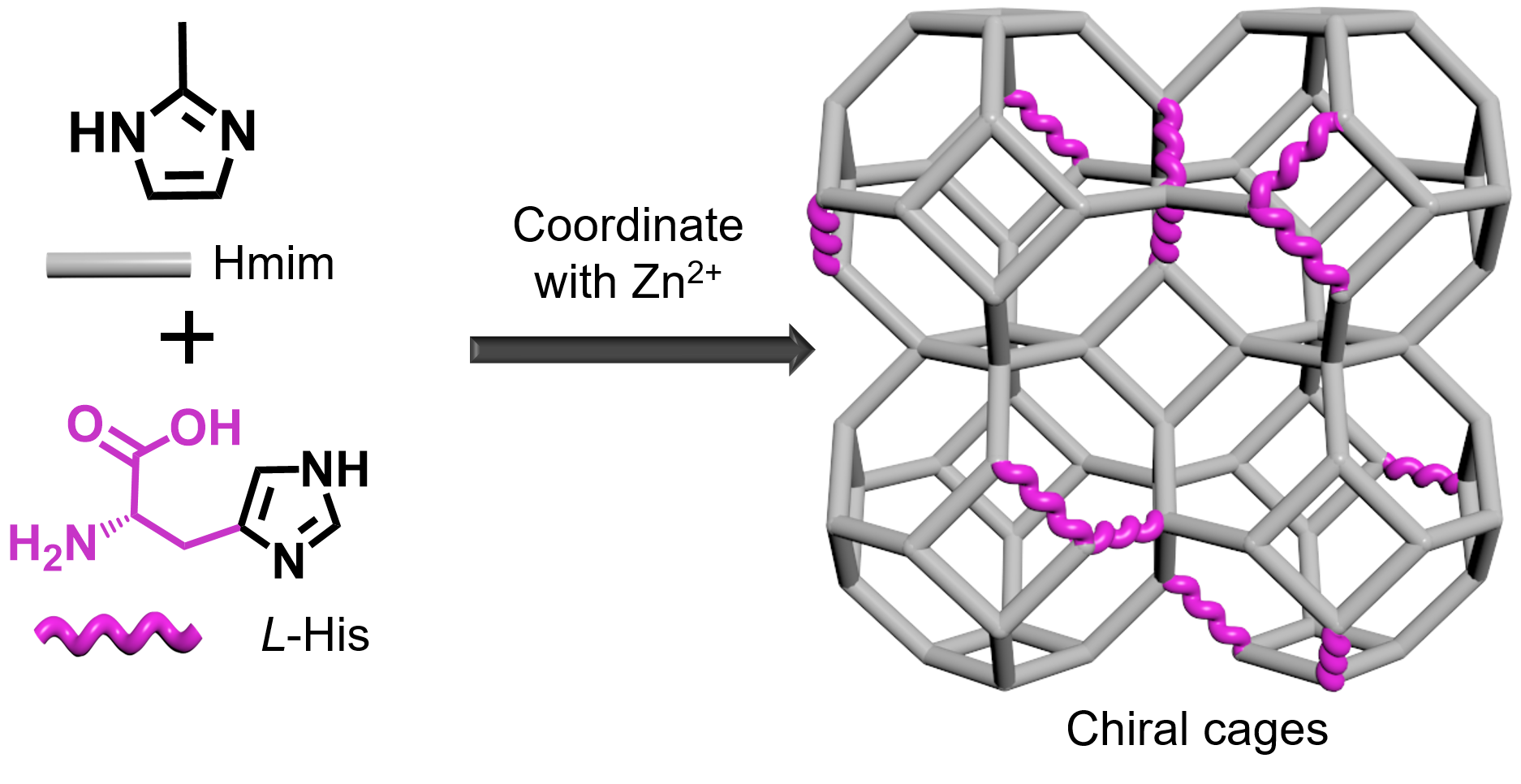


**Figure S5.** Schematic representation of the synthetic of chiral cages.


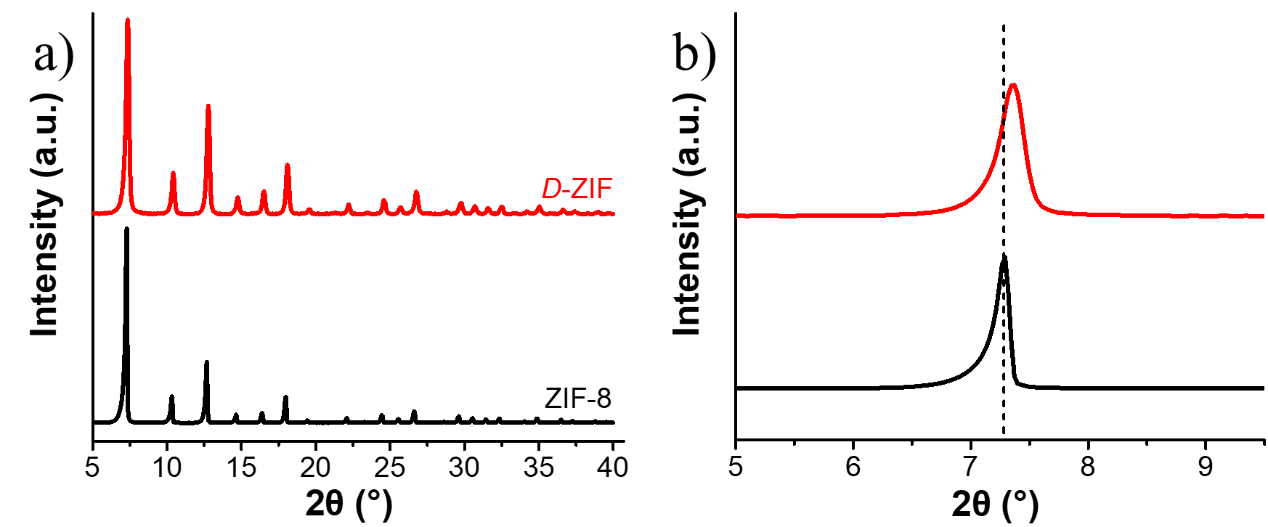


**Figure S6.** a) PXRD patterns of *D*-ZIF and ZIF-8. b) Enlarged PXRD patterns of *D*-ZIF and ZIF-8 at first-order diffraction peak.

**
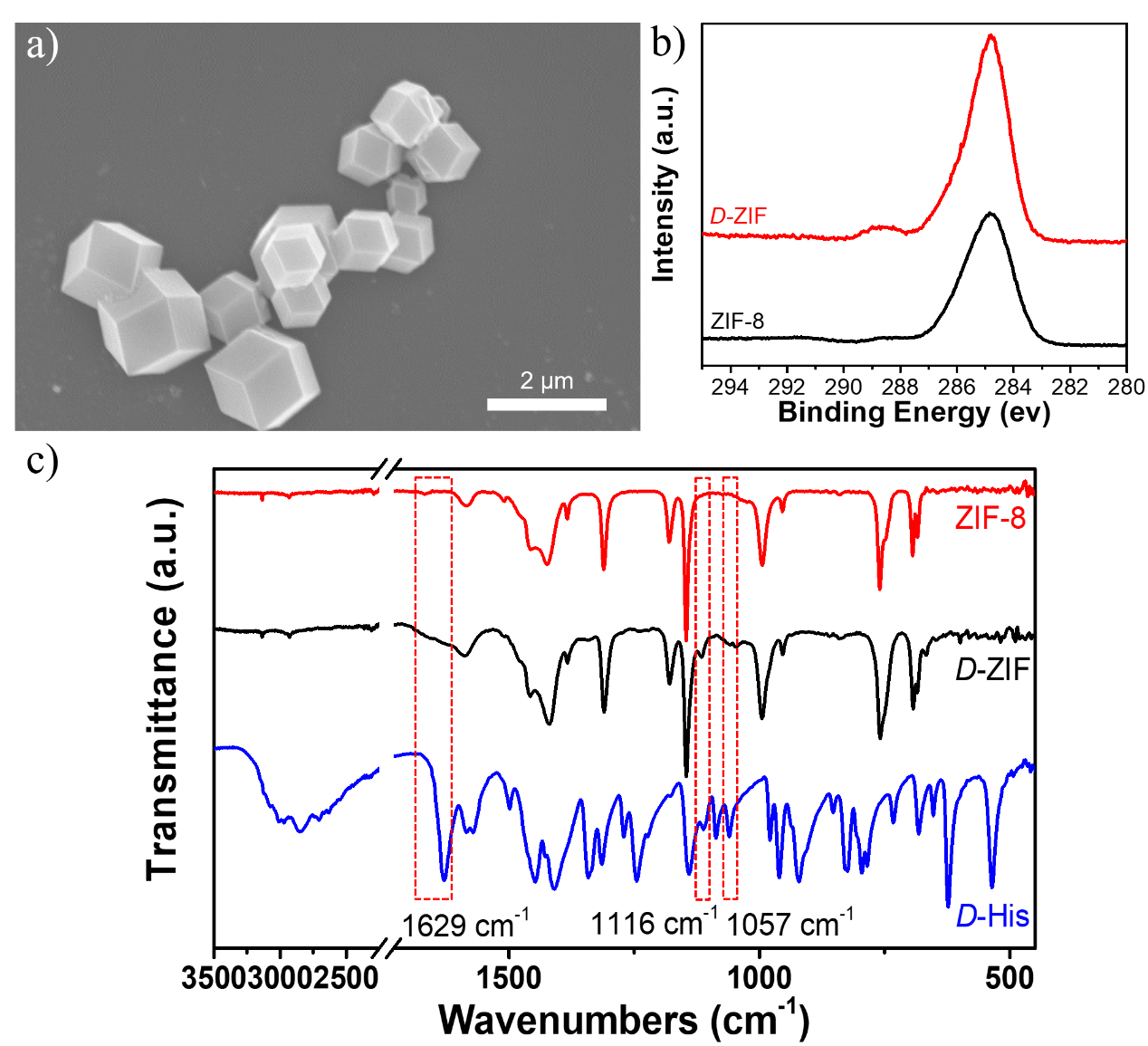
**

**Figure S7.** a) SEM image of D-ZIF. b) XPS analysis of C1s between D-ZIF and ZIF-8 crystals. c) FT-IR spectra of D-ZIF, D-His and ZIF-8.

**
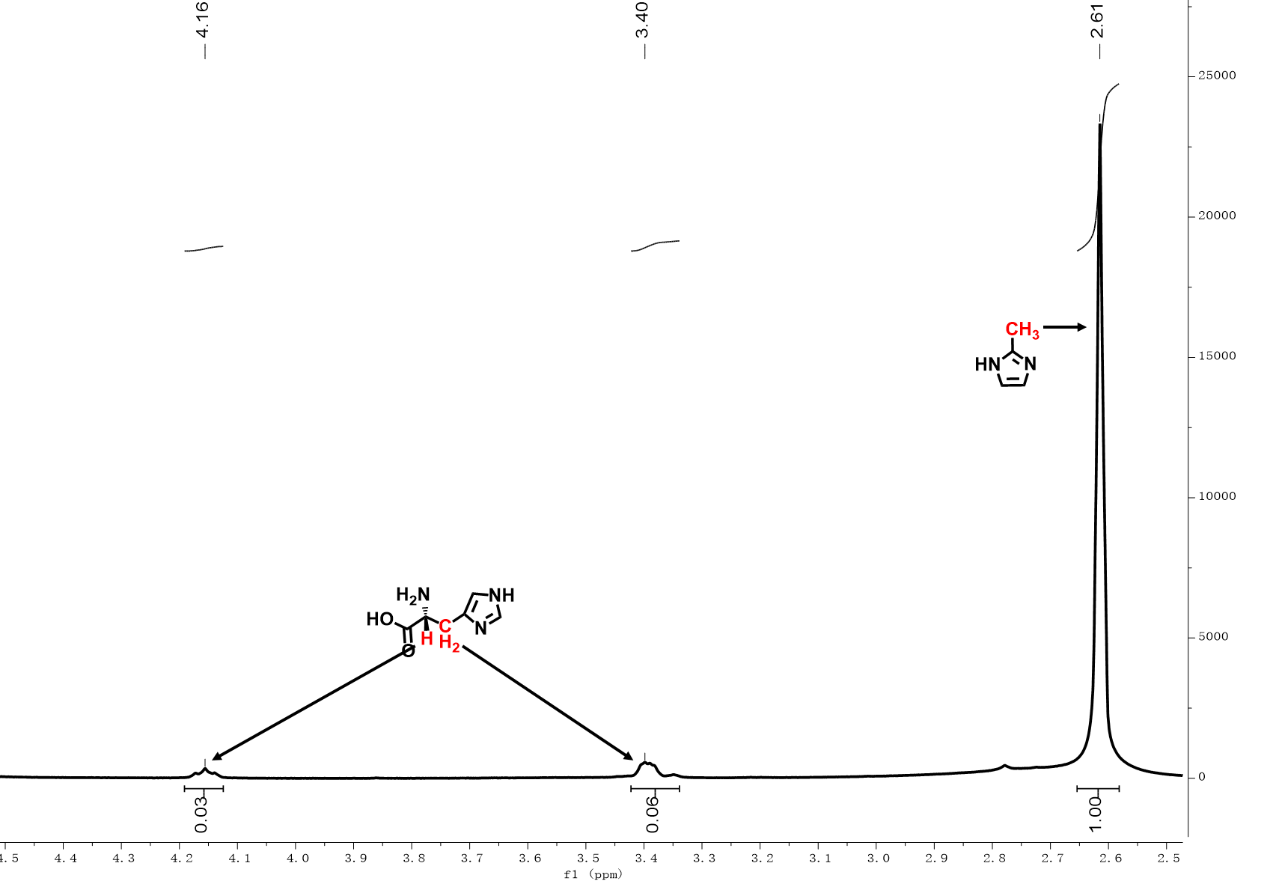
**

**Figure S8.** Solution ^1^H-NMR of acid-digested D-ZIF obtained from n_Hmim_/n_His_ = 7/1 during the synthesized process.


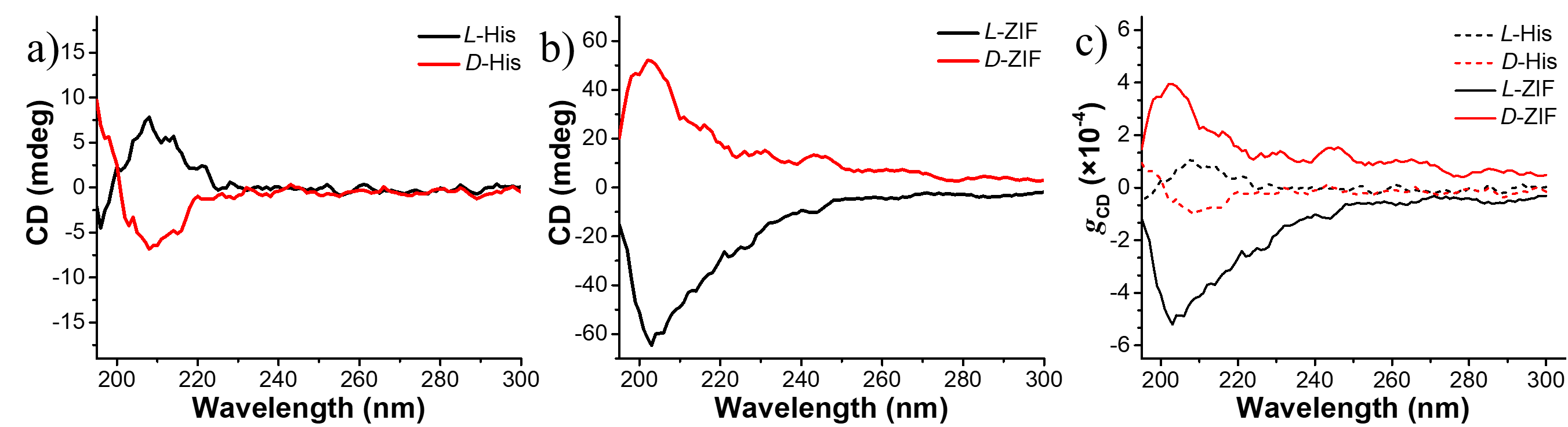


**Figure S9.** CD spectra of a) L-/D-histidine (1.5 × 10^-3^ M) in methanol and b) L-/D-ZIF in methanol. c) CD dissymmetric factor *g*_CD_ as a function of the wavelength.

**S2. Synthesis and characterization of *L*-/D-ZIF loading with dyes**

(1) Synthesis of *L*-ZIF⊃dye (S420 or C6 or DCM):

A mixture of dye (0.03 mmol), 2-methylimidazole (260 mg, 3.15 mmol) and L-histidine (70 mg, 0.45 mmol) was dissolved in 15 mL mixed solution of H_2_O/methanol (2:3 v/v) equipped with a magnetic stirring bar. Then 60 μL triethylamine was added followed by stirring for 10 min. After that, the mixed-ligand solution was gradually added to the methanol solution (15 mL) of Zn(NO_3_)_2_•6H_2_O (270 mg, 0.9 mmol). The reaction was carried out stirring at room temperature for 24h. The resulting product was collected by centrifugation and repeatedly washed with 30 mL H_2_O/methanol (2:3 v/v) four times. The collected powder was dried in vacuum. It should be mentioned that due to the less solubility of C6 and DCM in H_2_O, the resulting product was washed with 30 mL N,N-dimethyl formamide (DMF) for two times firstly, then washed by 30 mL methanol three times. After that, the collected powder was dried in vacuum.

D-ZIF⊃dye was synthesized as same as L-ZIF⊃dye excepted D-histidine was instead of L-histidine.

(2) Synthesis of *L*-ZIF⊃S420/C6/DCM:

A mixture of S420 (1 mg, 0.0017 mmol), C6 (6 mg, 0.02 mmol), DCM (6 mg, 0.02 mmol), 2-methylimidazole (260 mg, 3.15 mmol) and L-histidine (70 mg, 0.45 mmol) was dissolved in 15 mL mixed solution of H_2_O/methanol (2:3 v/v) equipped with a magnetic stirring bar. Then 60 μL triethylamine was added followed by stirring for 10 min. After that, the mixed-ligand solution was gradually added to the methanol solution (15 mL) of Zn(NO_3_)_2_•6H_2_O (270 mg, 0.9 mmol). The reaction was carried out stirring at room temperature for 24h. The resulting product was collected by centrifugation and repeatedly washed with 30 mL DMF two times. Then washed with 30 mL methanol three times. The collected powder was dried in vacuum.

D-ZIF⊃ S420/C6/DCM was synthesized as same as L-ZIF excepted D-histidine was instead of L-histidine.

(3) Synthesis of ZIF-8⊃DCM:

A mixture of 2-methylimidazole (300 mg, 3.6 mmol) and DCM (9.2 mg, 0.03 mmol) was dissolved in 15 mL methanol. Then, the solution was gradually added to the methanol solution (15 mL) of Zn(NO_3_)_2_•6H_2_O (270 mg, 0.9 mmol). The reaction was carried out stirring at room temperature for 24h. The resulting product was collected by centrifugation and repeatedly washed with methanol four times. The collected orange-yellow powder was dried in vacuum.

**Determination of dye contents.** The fluorescence intensity of different concentrations of S420, C6 and DCM in methanol from 2 × 10^-7^ to 2 × 10^-6^ were measured and repeated five times.^[1]^ The relationship for the intensity-concentration of various dyes was obtained (Figure S25-S27). Well-dried chiral ZIF⊃dye powder (~15 mg) was redispersed in 5 mL methanol, then moderate diluted hydrochloric acid was added to decompose ZIF powder. After removed solvent and hydrochloric acid, the residuum was resolved in 20 to 50 mL methanol and then the luminescent intensity of the solutions was measured. The concentrations of S420, C6 and DCM were calculated through the intensity-concentration equation in Figure S25-S27, respectively.

S420: y = 4.997 × 10^8^ x + 76.88525;

C6: y = 2.694 × 10^9^ x + 47.79672;

DCM: y = 1.015 × 10^9^ x + 35.81967;

y - fluorescence intensity;

x – concentration of dye.

**
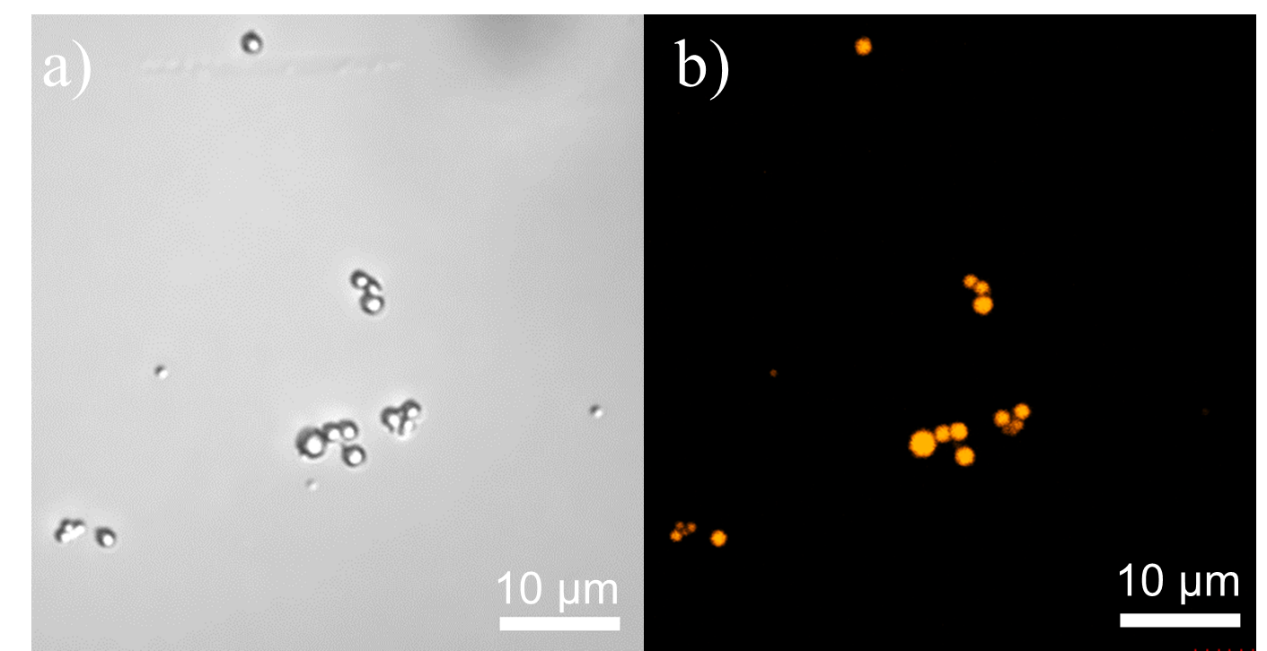
**

**Figure S10.** a) Optical microscopy images and b) laser scanning confocal microscopy images obtained from L-ZIF⊃DCM (0.04 wt%), λ_ex_ = 405 nm.


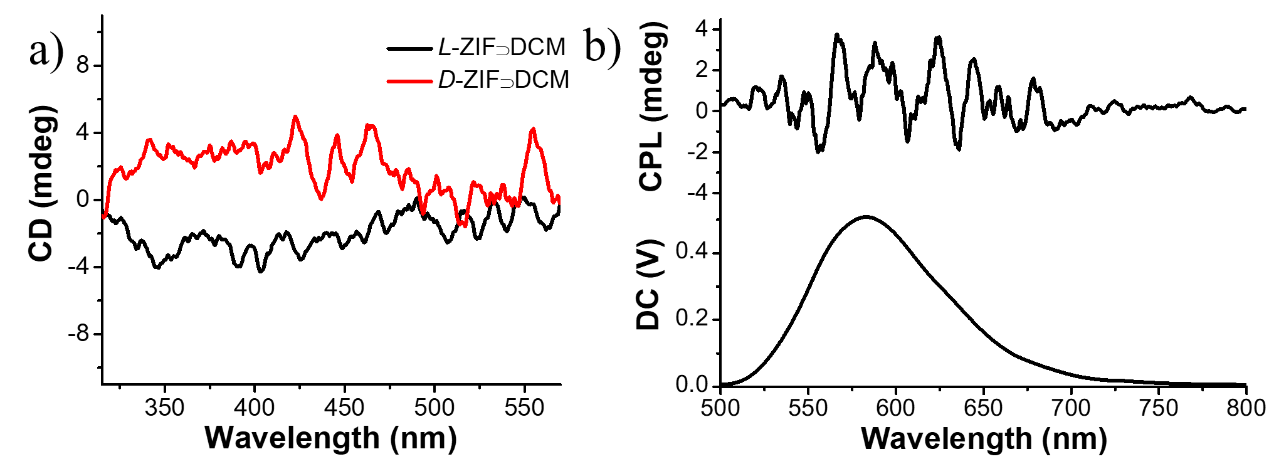


**Figure S11.** a) CD spectra of L-/D-ZIF⊃DCM (0.04 wt %) in methanol. b) CPL spectra of ZIF-8⊃DCM, λ_ex_ = 450 nm.

**
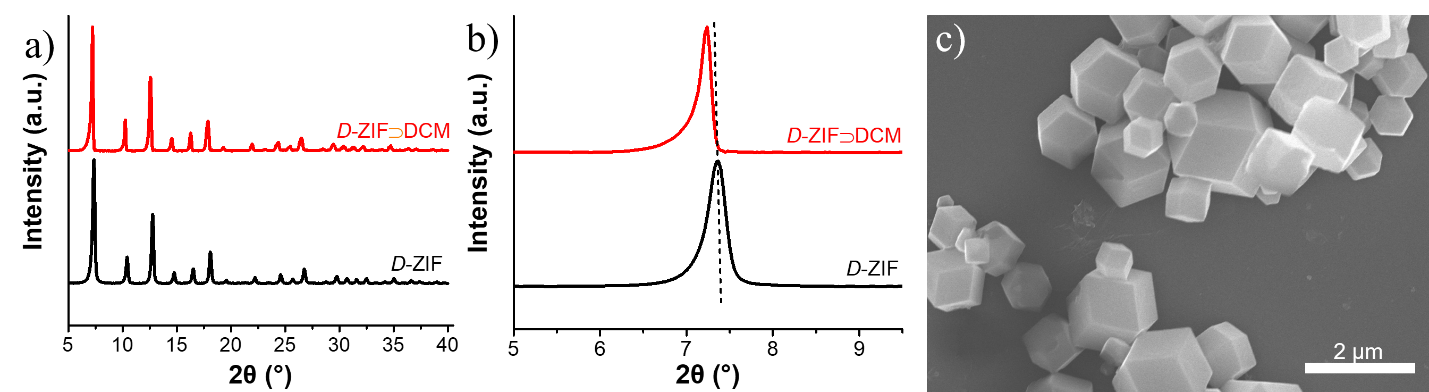
**

**Figure S12.** a) XRD patterns of *D*-ZIF and *D*-ZIF⊃DCM (0.04 wt%). b) Enlarged XRD patterns of *D*-ZIF and *D*-ZIF⊃DCM (0.04 wt%) at first-order diffraction peak. c) SEM image of *D*-ZIF⊃DCM (0.04 wt%).


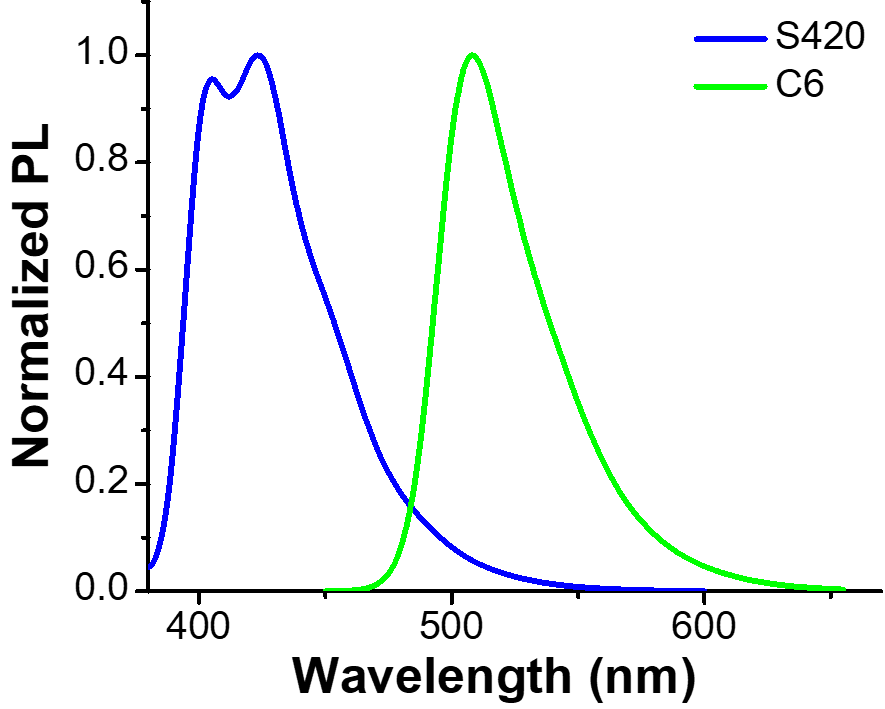


**Figure S13.** Normalized fluorescence spectra of S420 **(**1 × 10^-5^ M, λ_ex_ = 360 nm) and C6 **(**1 × 10^-5^ M, λ_ex_ = 450 nm) in methanol.


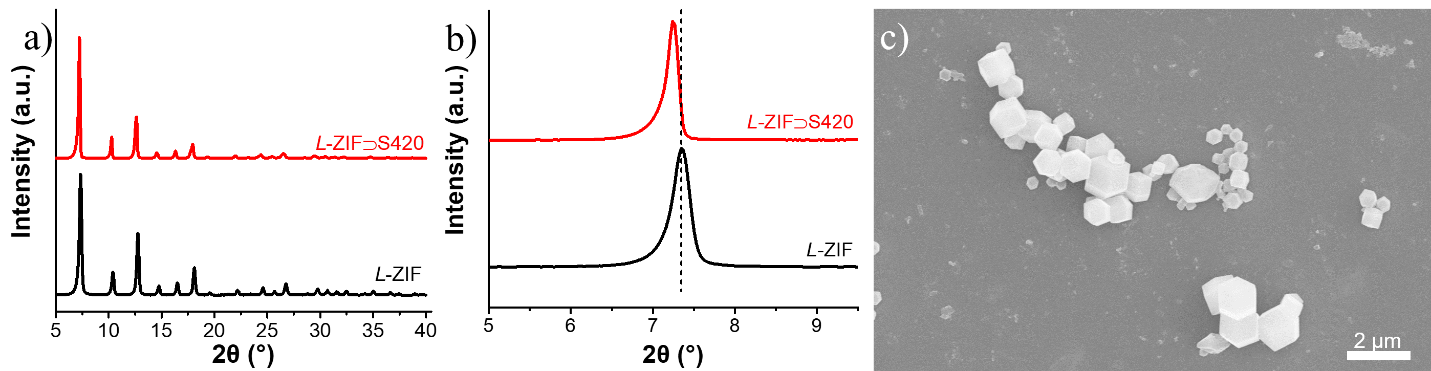


**Figure S14.** a) XRD patterns of *L*-ZIF and *L*-ZIF⊃S420 (0.3 wt%). b) Enlarged XRD patterns of *L*-ZIF and *L*-ZIF⊃S420 (0.3 wt%) at first-order diffraction peak. c) SEM image of *L*-ZIF⊃S420 (0.3 wt%).


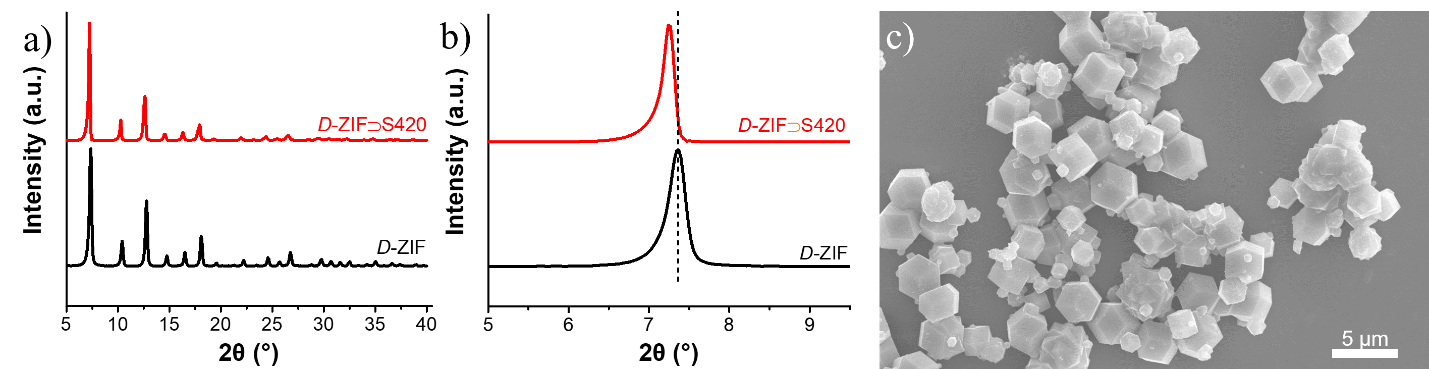


**Figure S15.** a) XRD patterns of *D*-ZIF and *D*-ZIF⊃S420 (0.3 wt%). b) Enlarged XRD patterns of *D*-ZIF and *D*-ZIF⊃S420 (0.3 wt%) at first-order diffraction peak. c) SEM image of *D*-ZIF⊃S420 (0.3 wt%).


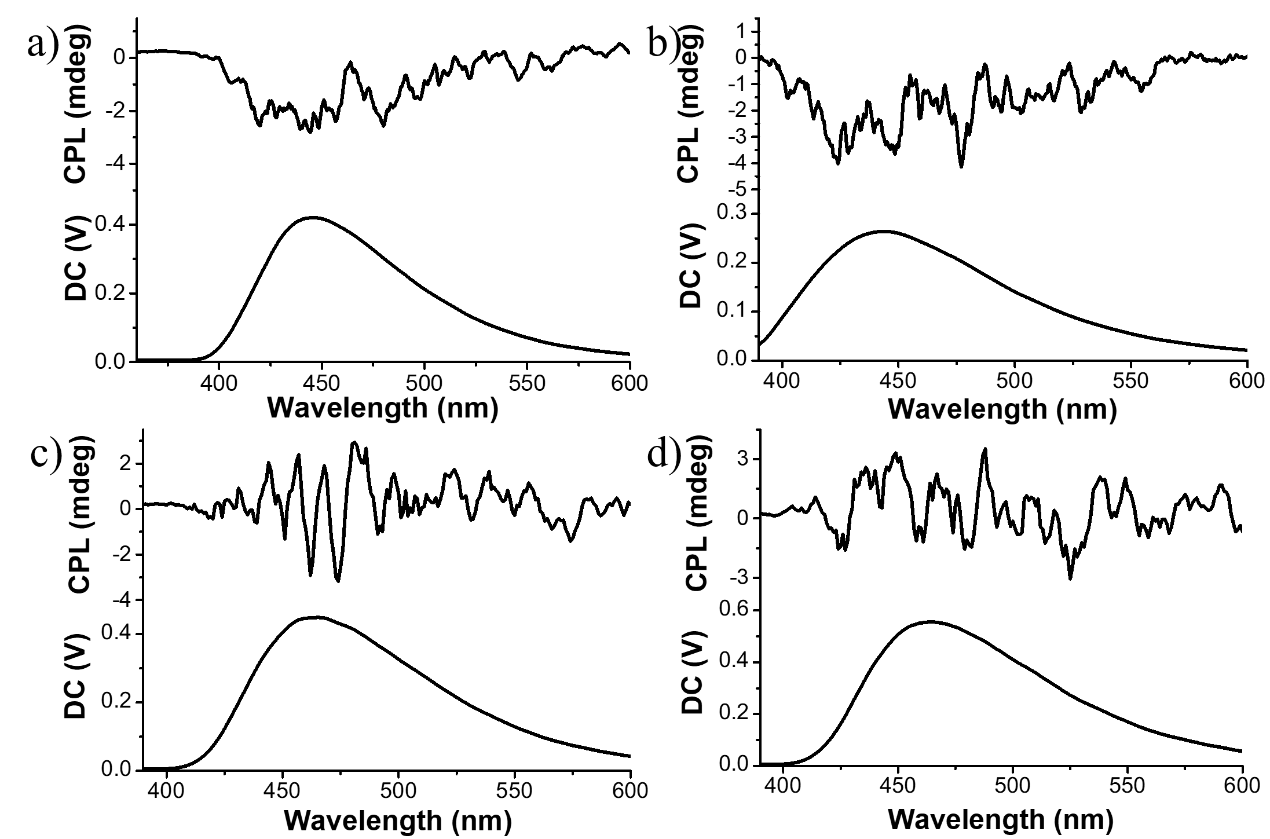


**Figure S16.** CPL spectra of *L*-ZIF⊃S420 with the content of S420 was a) 0.015 mmol, b) 0.03 mmol, c) 0.06 mmol, d) 0.09 mmol during the synthesized process.


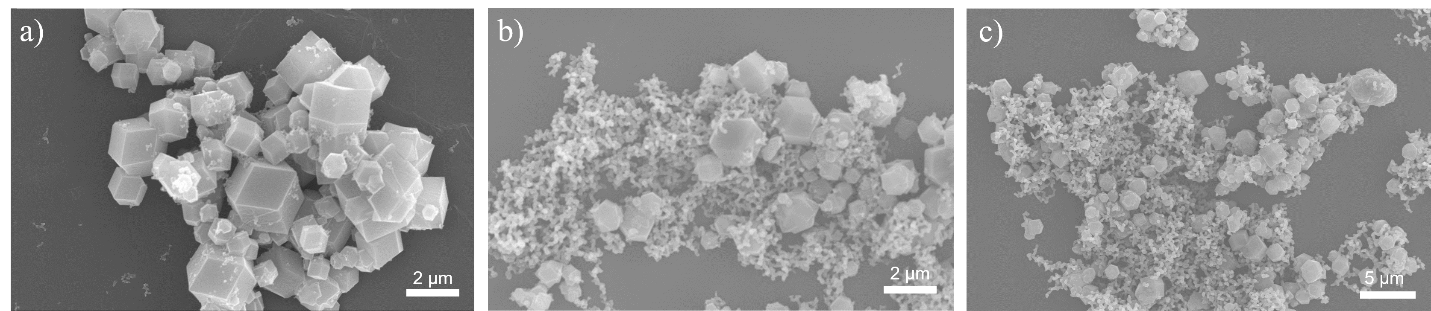


**Figure S17.** SEM images of *L*-ZIF⊃S420 with the content of S420 was a) 0.015 mmol, b) 0.06 mmol, c) 0.09 mmol during the synthesized process.


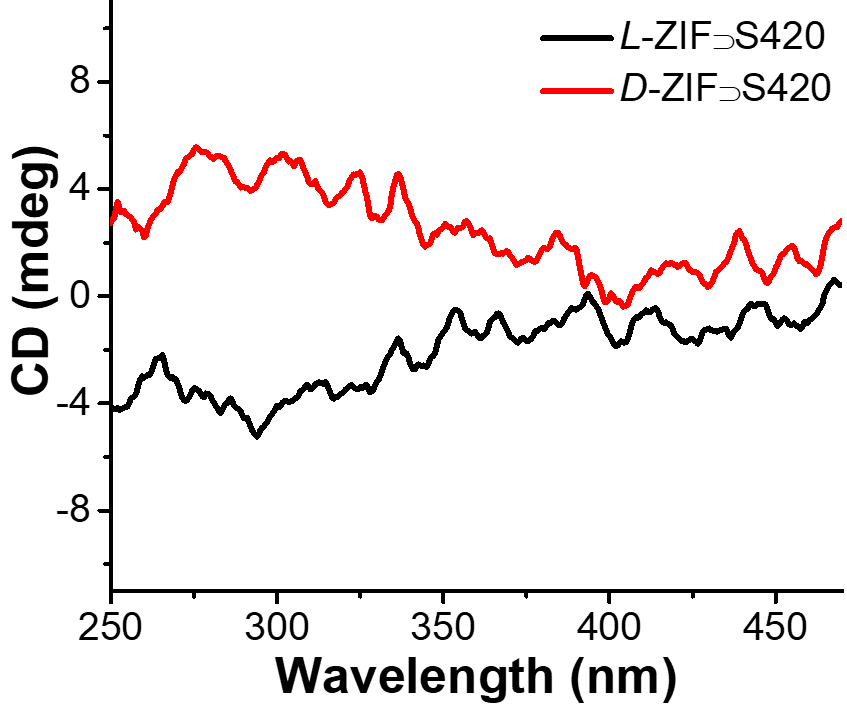


**Figure S18.** CD spectra of L-/D-ZIF⊃S420 (0.3 wt %) in methanol.


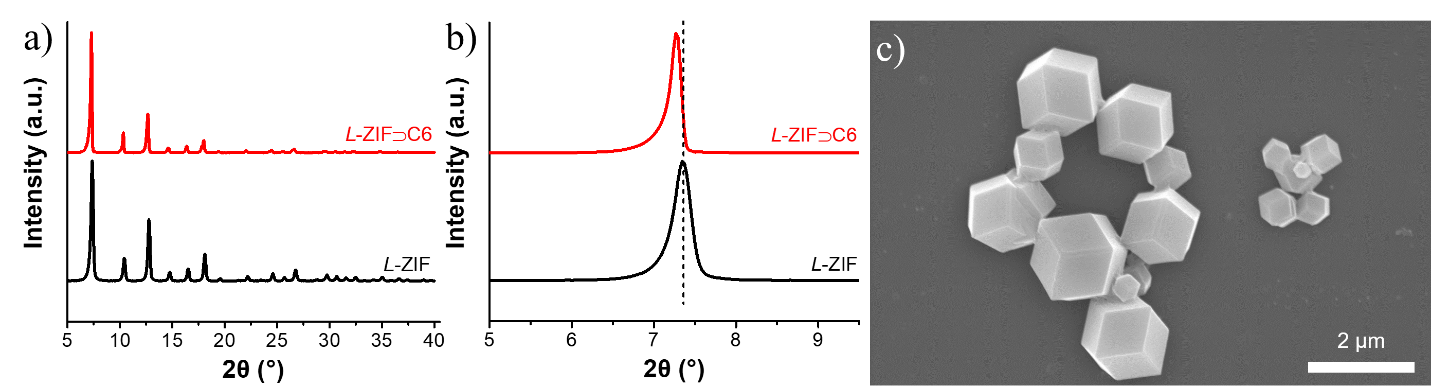


**Figure S19.** a) XRD patterns of *L*-ZIF and *L*-ZIF⊃C6 (0.04 wt%). b) Enlarged XRD patterns of *L*-ZIF and *L*-ZIF⊃C6 (0.04 wt%) at first-order diffraction peak. c) SEM image of *L*-ZIF⊃C6 (0.04 wt%).


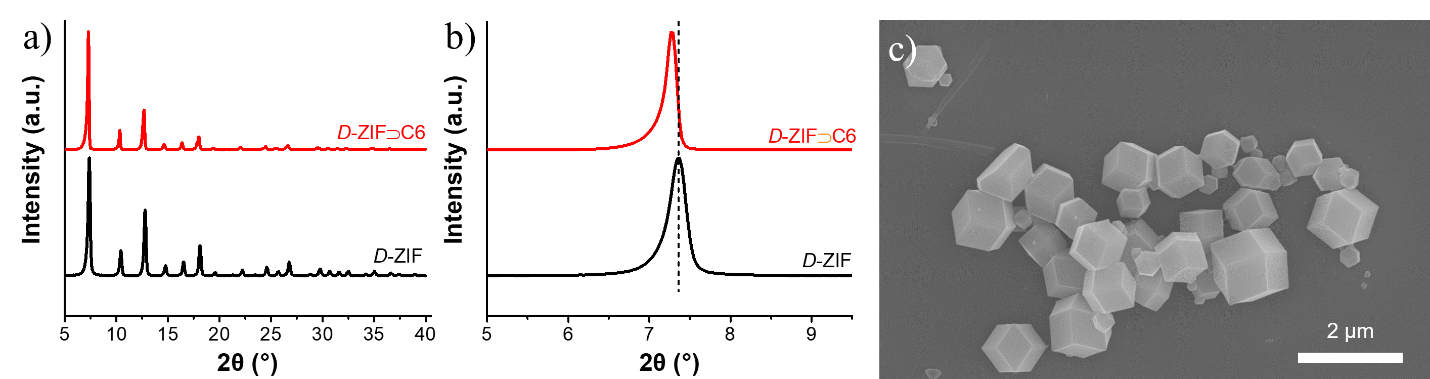


**Figure S20.** a) XRD patterns of *D*-ZIF and *D*-ZIF⊃C6 (0.04 wt%). b) Enlarged XRD patterns of *D*-ZIF and *D*-ZIF⊃C6 (0.04 wt%) at first-order diffraction peak. c) SEM image of *D*-ZIF⊃C6 (0.04 wt%).


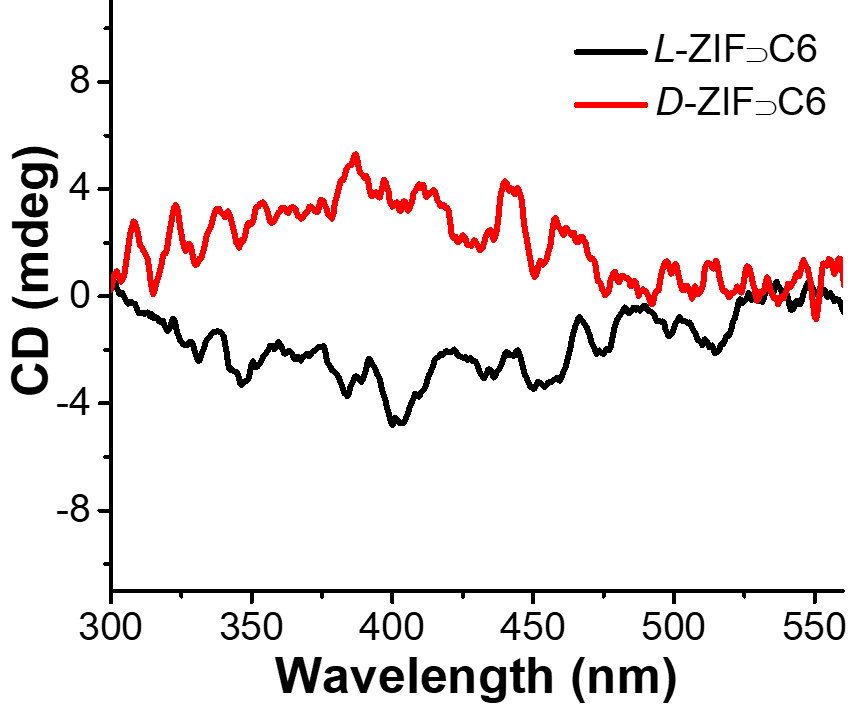


**Figure S21.** CD spectra of L-/D-ZIF⊃C6 (0.04 wt%) in methanol.


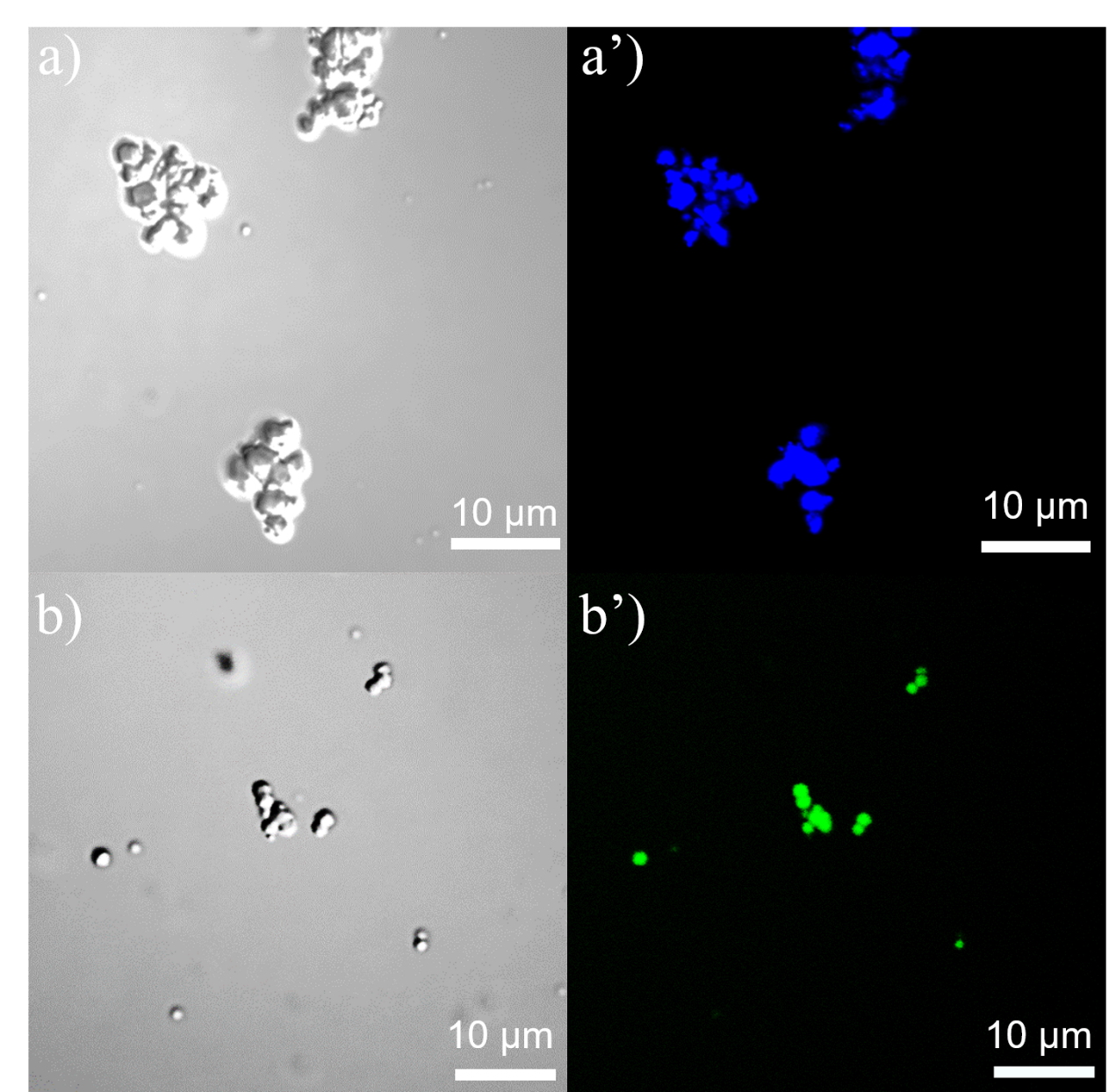
**Figure S22.** a, b) Optical microscopy images and a’,b’) laser scanning confocal microscopy images made from L-ZIF⊃S420 (0.3 wt%) and *L*-ZIF⊃C6 (0.04 wt%), respectively, λ_ex_ = 405 nm.


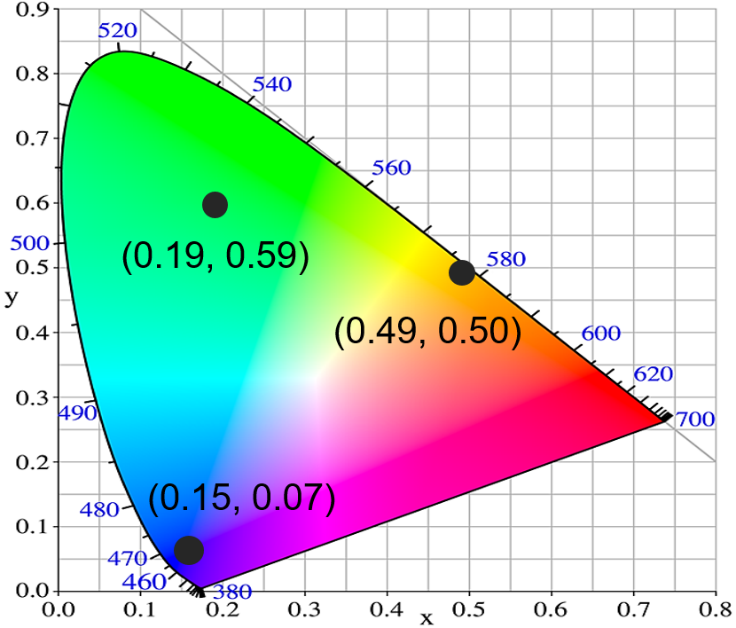


**Figure S23.** The CIE coordinates of *L*-ZIF⊃S420 (0.3 wt%), *L*-ZIF⊃C6 (0.04 wt%), and *L*-ZIF⊃DCM (0.04 wt%), λ_ex_ = 370 nm.

**Table S1.** Photophysical parameters of dyes and *L-*/*D*-ZIF⊃dyes in solid state.

|  | Powder | | |  | Powder | | | | | |
| --- | --- | --- | --- | --- | --- | --- | --- | --- | --- | --- |
|  | λ_em_  [nm] | Φ_PL_^c)^  [%] | τ  [ns] |  | λ_em_^d)^  [nm] | Φ_PL_^c)^  [%] (*L*-ZIF) | τ  [ns] (*L*-ZIF) | Φ_PL_^c)^  [%] (*D*-ZIF) | τ  [ns] (*D*-ZIF) | *g*_lum_ (×10^-3^) |
| S420 ^a)^ | 477 | 37 | 4.6^e)^ |  | 426 | 59 | 2.2 | 58 | 2.3 | ±0.9 |
| C6^b)^ | 578 | 11 | 4.3 ^e)^ |  | 505 | 75 | 2.4 | 76 | 2.4 | ±0.3 |
| DCM^b)^ | 640 | 2 | 1.8 ^e)^ |  | 578 | 43 | 2.3 | 37 | 2.3 | ±1.2 |

a) Excitation by 360 nm; b) Excitation by 450 nm; c) Absolute quantum yield; d) Fluorescence of the dyes encapsulated in chiral ZIFs; e) Double-exponential fit, and fluorescence lifetime calculated using the equation τ = A_1_τ_1_ + A_2_τ_2_.


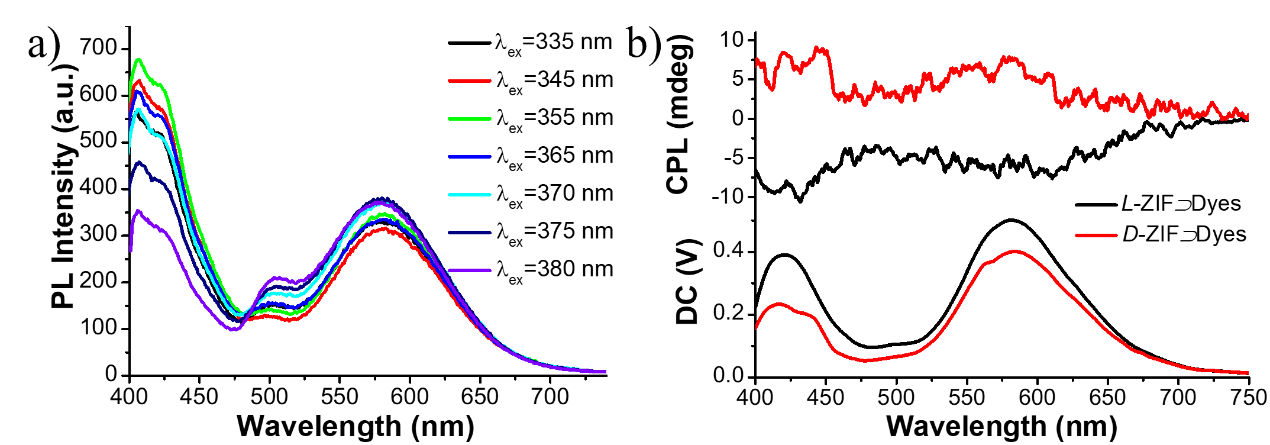


**Figure S24.** a) Fluorescence spectra of L-ZIF⊃S420/C6/DCM (0.02wt% S420, 0.03wt% C6, 0.03wt% DCM) with excitation wavelengths varied from 335 to 380 nm. b) CPL spectra of L-/D-ZIF⊃S420/C6/DCM (0.02wt% S420, 0.03wt% C6, 0.03wt% DCM), λ_ex_ = 360 nm.


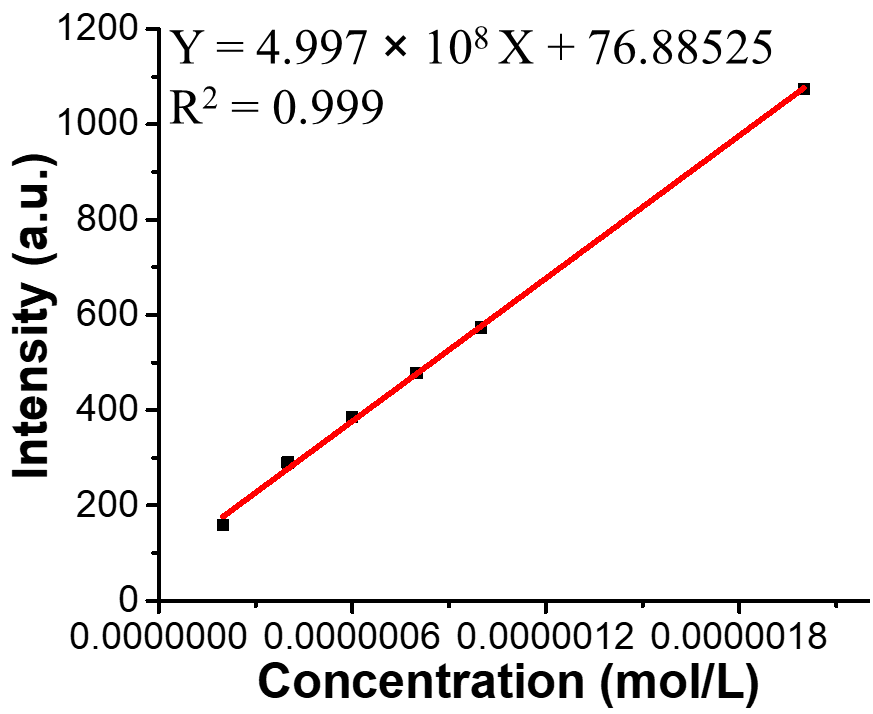


**Figure S25.** The intensity-concentration relationship for the methanol solution of S420.


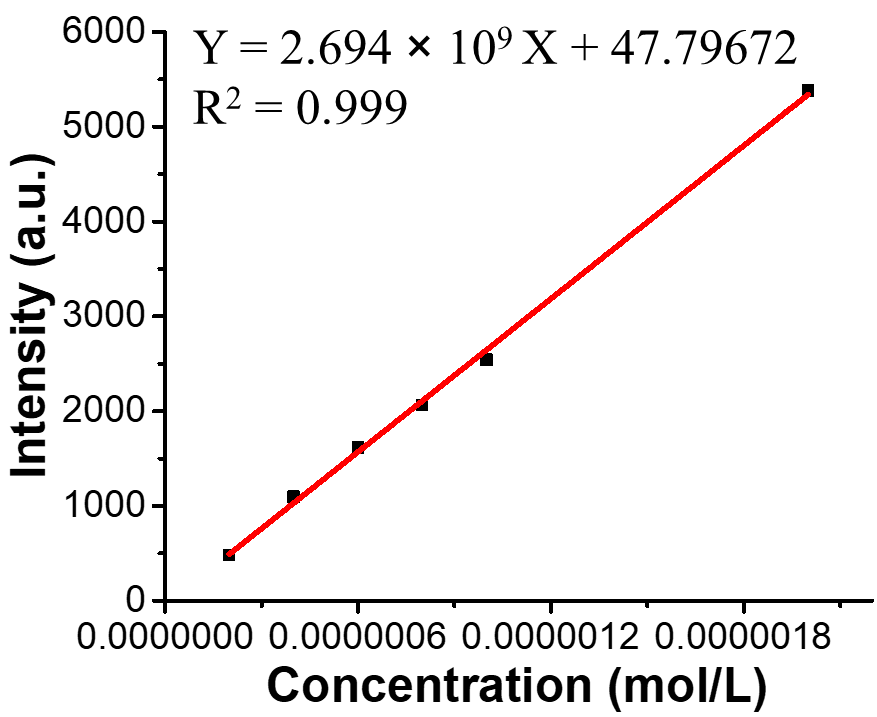


**Figure S26.** The intensity-concentration relationship for the methanol solution of C6.


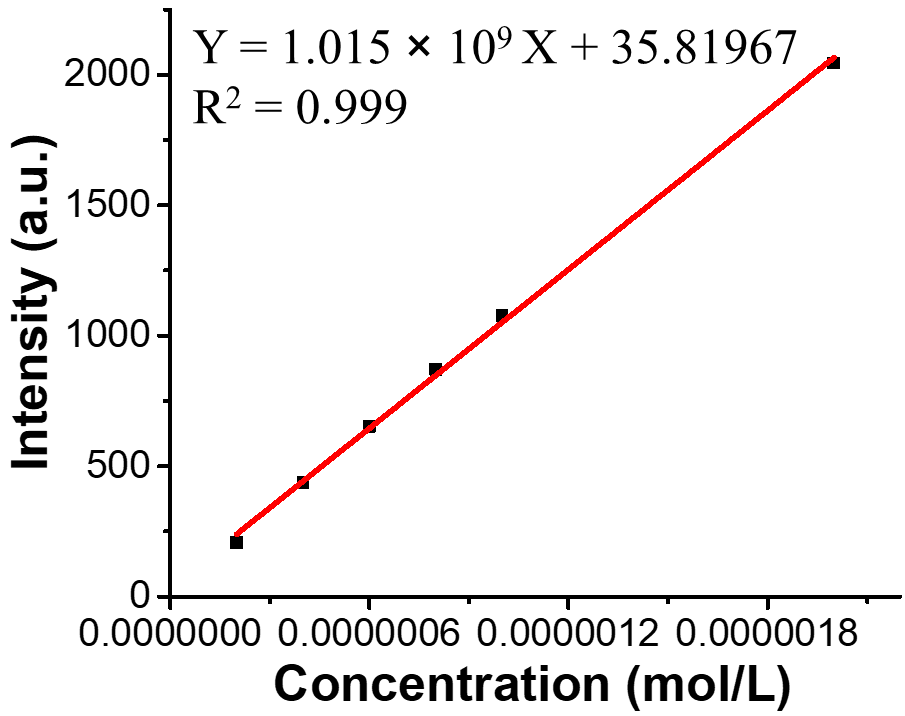


**Figure S27.** The intensity-concentration relationship for the methanol solution of DCM.

**Reference:**

[1] Y. J. Cui, T. Song, J. C. Yu, Y. Yang, Z. Y. Wang, G. D. Qian, *Adv. Funct. Mater.* **2015**, 25, 4796.

**S3. Synthesis and characterization of *L*-/D-ZIF loading with quantum dots (QDs)**

(1) PVP modification for all CdSe/ZnS QDs:

GdSe/ZnS QDs were dipersed in 20 ml of chloroform (0.5 mg/ml). A solution of PVP (62.5 mg, Mw = 10,000) in chloroform (10 ml) was then added. After the mixture was stirred for 24 hours, the PVP-modified QDs were precipitated with *n*-hexane and collected by centrifugation. The sample was cleaned with chloroform and hexane (1:1 v/v) to remove the excess free PVP. Finally, the PVP-modified QDs were redispersed in methanol.

(2) Synthesis of *L*-ZIF⊃QD composites:

A mixture of 2-methylimidazole (260 mg, 3.15 mmol) and L-histidine (70 mg, 0.45 mmol) was dissolved in 15 mL mixed solution of H_2_O/methanol (2:3 v/v) equipped with a magnetic stirring bar. Then 60 μL triethylamine was added followed by stirring for 10 min. After that, the mixed-ligand solution was gradually added to the methanol solution (15 mL) of Zn(NO_3_)_2_•6H_2_O (270 mg, 0.9 mmol) and QDs (0.3 mg/mL). The reaction was carried out stirring at room temperature for 24h. The resulting product was collected by centrifugation and repeatedly washed with 30 mL methanol four times. The collected powder was dried in vacuum. For the white-light emitting *L*-ZIF⊃QDs, a mixture of various QDs (0.3 mg/mL, the mass ratio of QD463, QD501, QD533, QD604 and QD647 was 6:1.5:3:2:1) was added.

D-ZIF⊃QD composites was synthesized as same as L-ZIF⊃QD excepted D-histidine was instead of L-histidine.


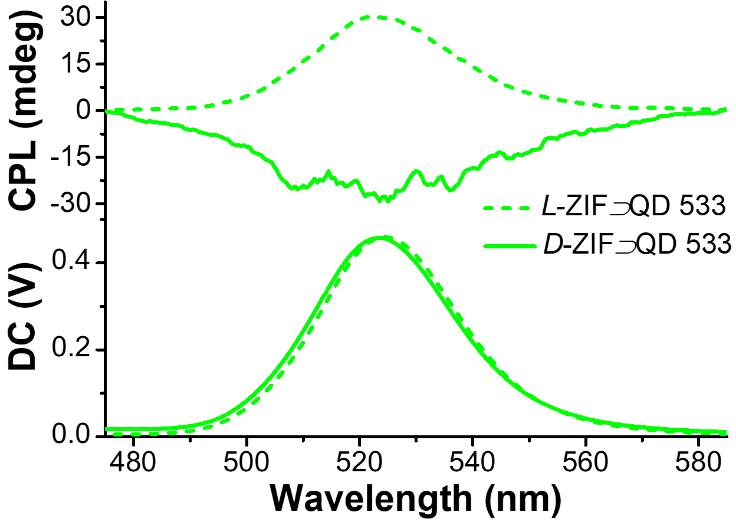


**Figure S28.** CPL spectra of *L*-ZIF⊃QD533 and *D*-ZIF⊃QD533 in solid state, λ_ex_ = 360 nm.


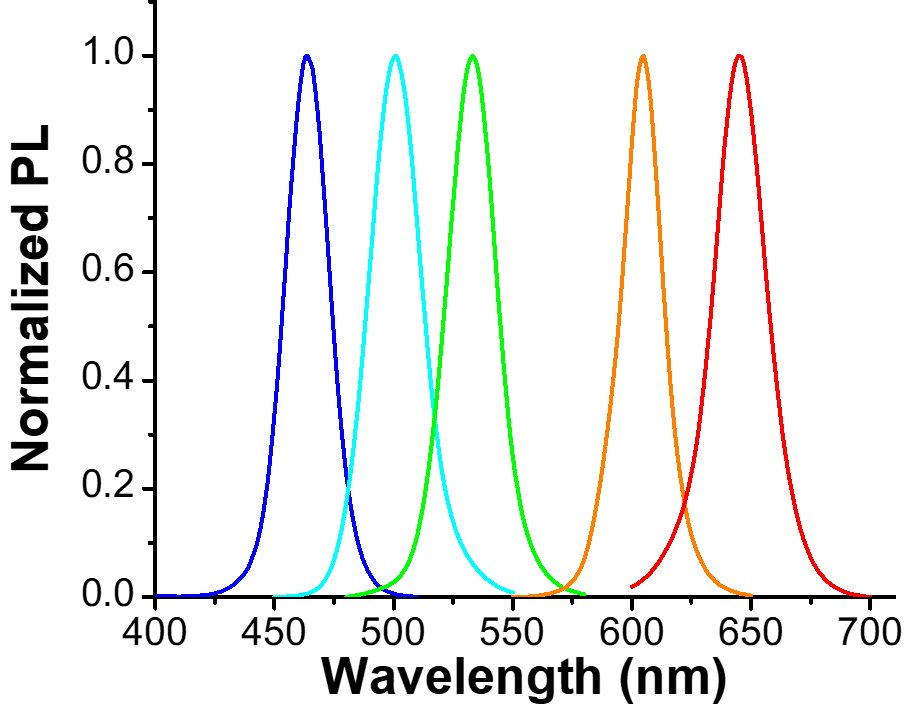


**Figure S29.** Normalized fluorescence spectra of various PVP-modified QDs in methanol (0.3 mg/mL).


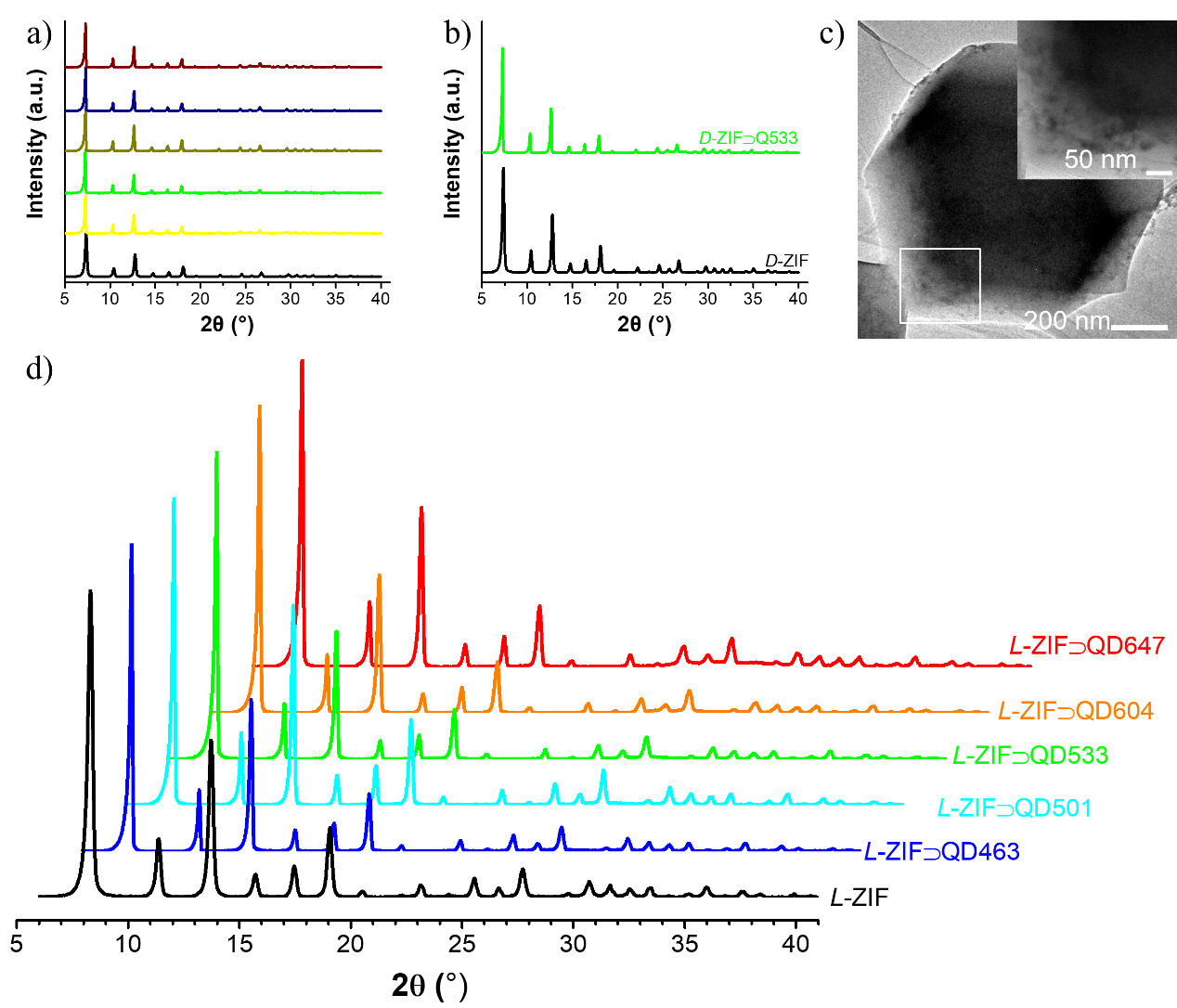


**Figure S30.** a) PXRD patterns of L-ZIF (black line) and L-ZIF⊃QD533 obtained from various concentration of QD533 used during the synthesized process (yellow line, 0.1 mg/mL; green line, 0.3 mg/mL; dark line, 0.5 mg/mL; navy line, 0.75 mg/mL; wine line, 1 mg/mL). b) PXRD patterns of D-ZIF (black line) and D-ZIF⊃QD533 (green line). c) TEM images of D-ZIF⊃QD533. d) PXRD patterns of L-ZIF and various L-ZIF⊃QD composites.

**Table S2.** Circularly polarized luminescence *g*_lum_ of *L-*/*D*-ZIF⊃QD composites in solid state.

|  | λ_em_^a)^  [nm] | *g*_lum_  (×10^-3^) | |
| --- | --- | --- | --- |
|  |  | *L*-ZIF | *D*-ZIF |
| QD463 | 463 | 3.0 | -2.7 |
| QD501 | 501 | 4.3 | -3.2 |
| QD533 | 533 | 4.6 | -4.3 |
| QD604 | 604 | 3.8 | -3.4 |
| QD647 | 647 | 3.0 | -3.2 |

a) Fluorescence of the QDs encapsulated in chiral ZIFs excited by 360 nm.


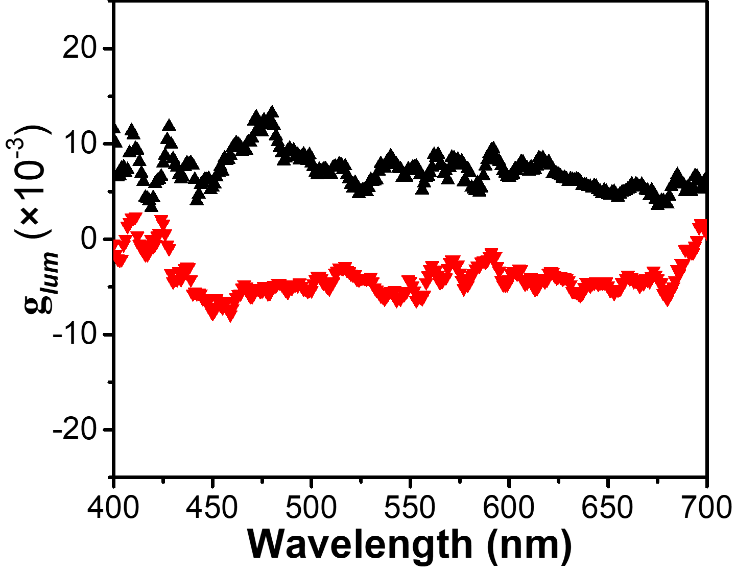


**Figure S31.** CPL dissymmetric factor *g*_lum_ as a function of the wavelength (L-ZIF⊃QDs: black; D-ZIF⊃QDs: red), λ_ex_ = 360 nm.

**S4. Synthesis and characterization of *L*-/D-ZIF loading with upconversion nanoparticles (UCNPs)**

(1) Synthesis of NaYF_4_:Yb, Er nanoparticles:

The NaY F_4_: 20% Yb, 2% Er UCNPs were prepared using a high-temperature co-precipitation method.^[1]^

(2) PVP modification for NaFY_4_:Yb, Er nanoparticles:

NaYF_4_:Yb, Er nanoparticles were dipersed in 20 ml of chloroform (0.5 mg/ml). A solution of PVP (62.5 mg, Mw = 10,000) in chloroform (10 ml) was then added. After the mixture was stirred for 24 hours, the PVP-modified UCNPs were precipitated with *n*-hexane and collected by centrifugation. The sample was cleaned with chloroform and hexane (1:1 v/v) to remove the excess free PVP. Finally, the PVP-modified UCNPs were redispersed in methanol (0.3 mg/mL).

(3) Synthesis of *L*-ZIF⊃UCNP composites:

A mixture of 2-methylimidazole (260 mg, 3.15 mmol) and L-histidine (70 mg, 0.45 mmol) was dissolved in 15 mL mixed solution of H_2_O/methanol (2:3 v/v) equipped with a magnetic stirring bar. Then 60 μL triethylamine was added followed by stirring for 10 min. After that, the mixed-ligand solution was gradually added to the methanol solution (15 mL) of Zn(NO_3_)_2_•6H_2_O (270 mg, 0.9 mmol) and UCNPs (0.3 mg/mL). The reaction was carried out stirring at room temperature for 24h. The resulting product was collected by centrifugation and repeatedly washed with 30 mL methanol four times. The collected precipitates was redispersed in methanol.

D-ZIF⊃UCNP composites was synthesized as same as L-ZIF⊃UCNP excepted D-histidine was instead of L-histidine.


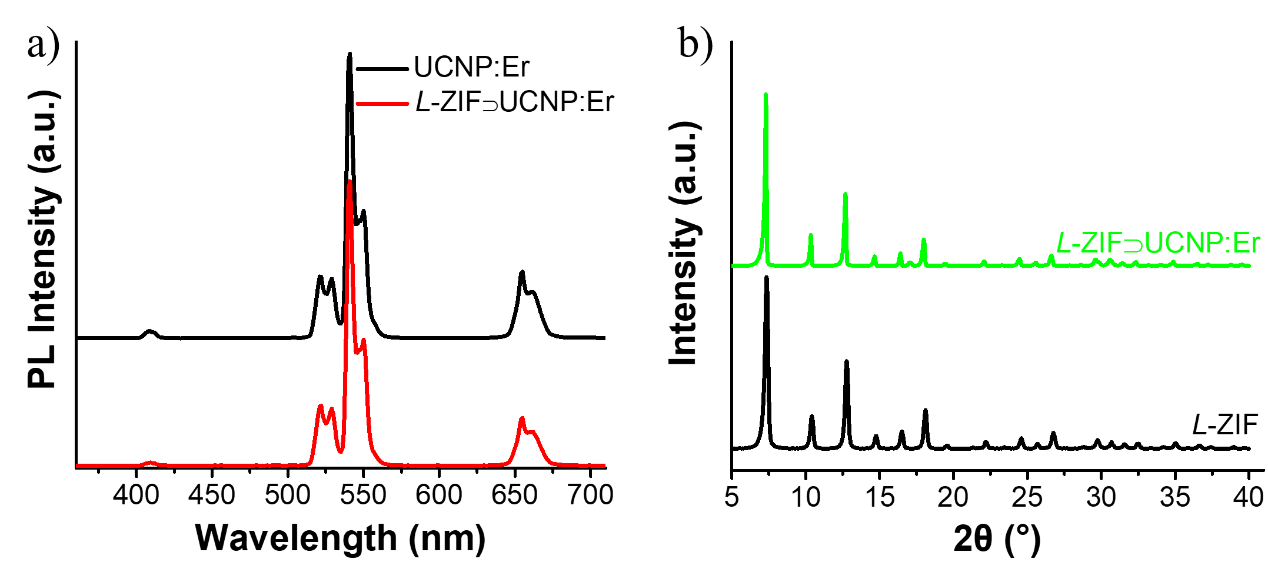


**Figure S32.** a) Upconverted luminescence spectra of UCNP:Er in methanol (0.3 mg/mL) and L-ZIF⊃UCNP:Er under 980 nm laser excitation. b) PXRD patterns of L-ZIF and L-ZIF⊃UCNP:Er.


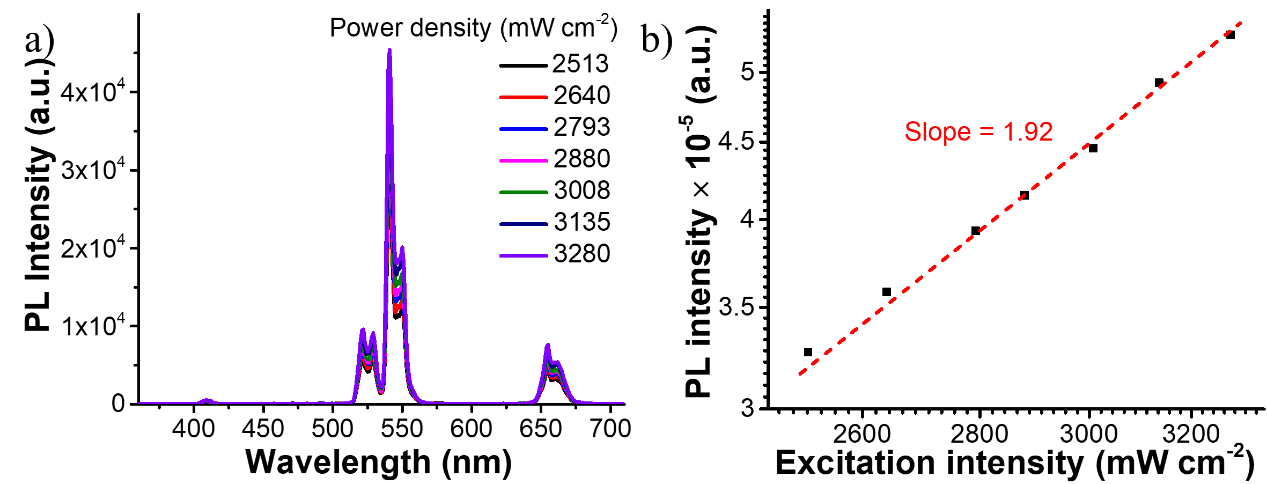


**Figure S33.** a) Upconverted luminescence spectra of L-ZIF⊃UCNP:Er composites with different incident power density of 980 nm laser. b) The double-logarithmic plots of the integrated UC emission intensity of L-ZIF⊃UCNP:Er composites as a function of excitation intensity of the 980 nm laser.


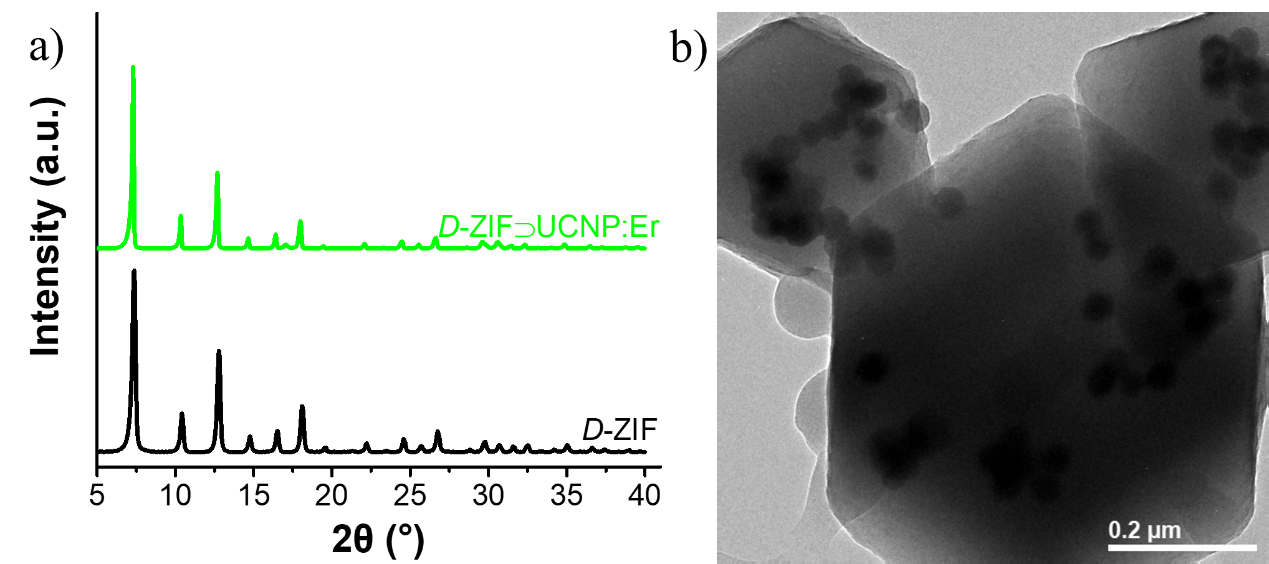


**Figure S34.** a) PXRD patterns of D-ZIF and D-ZIF⊃UCNP:Er. b) TEM image of D-ZIF⊃UCNP:Er.

**Table S3.** Circularly polarized luminescence *g*_lum_ of *L-*/*D*-ZIF⊃UCNP composites.

|  | λ_em_^a)^  [nm] | *g*_lum_  (×10^-2^) | |
| --- | --- | --- | --- |
|  |  | *L*-ZIF | *D*-ZIF |
| UCNP:Er | 409 | 1.2 | -1.3 |
|  | 522 | 1.2 | -1.4 |
|  | 541 | 1.1 | -1.0 |
|  | 655 | 1.2 | -1.0 |

a) Upconverted emission of the UCNP encapsulated in chiral ZIFs excited by 980 nm laser.


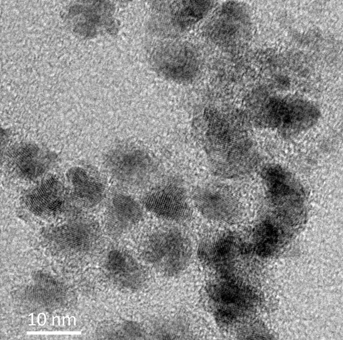


**Figure S35.** TEM image of PVP-modified QD533.

**Reference:**

[1] M. Zeng, S. Singh, Z. Hens, J. Liu, F. Artizzu, R. V. Deun, *J. Mater. Chem. C* **2019**, 7, 2014.
